# Defective LAT signalosome pathology in mice mimics human IgG4-related disease at single-cell level

**DOI:** 10.1084/jem.20231028

**Published:** 2023-08-25

**Authors:** Anais Joachim, Rudy Aussel, Léna Gélard, Fanghui Zhang, Daiki Mori, Claude Grégoire, Sergio Villazala Merino, Mauro Gaya, Yinming Liang, Marie Malissen, Bernard Malissen

**Affiliations:** 1 Aix Marseille Université, INSERM, CNRS, Centre d’Immunologie de Marseille-Luminy, Marseille, France; 2 Centre d’Immunophénomique, INSERM, CNRS, Aix Marseille Université, Marseille, France; 3 School of Laboratory Medicine, Henan Key Laboratory for Immunology and Targeted Therapy, Xinxiang Medical University, Xinxiang, China; 4 Laboratory of Immunophenomics, School of Laboratory Medicine, Xinxiang Medical University, Xinxiang, China

## Abstract

Mice with a loss-of-function mutation in the LAT adaptor (*Lat*^Y136F^) develop an autoimmune and type 2 inflammatory disorder called defective LAT signalosome pathology (DLSP). We analyzed via single-cell omics the trajectory leading to *Lat*^Y136F^ DLSP and the underlying CD4^+^ T cell diversification. T follicular helper cells, CD4^+^ cytotoxic T cells, activated B cells, and plasma cells were found in *Lat*^Y136F^ spleen and lung. Such cell constellation entailed all the cell types causative of human IgG4-related disease (IgG4-RD), an autoimmune and inflammatory condition with *Lat*^Y136F^ DLSP-like histopathological manifestations. Most previously described T cell–mediated autoimmune manifestations require persistent TCR input. In contrast, following their first engagement by self-antigens, the autoreactive TCR expressed by *Lat*^Y136F^ CD4^+^ T cells hand over their central role in T cell activation to CD28 costimulatory molecules. As a result, all subsequent *Lat*^Y136F^ DLSP manifestations, including the production of autoantibodies, solely rely on CD28 engagement. Our findings elucidate the etiology of the *Lat*^Y136F^ DLSP and qualify it as a model of IgG4-RD.

## Teaser

Joachim et al. deciphered via single-cell transcriptomics and functional genomics the etiopathology of a fast-onset mouse autoimmune and inflammatory disorder due to defective LAT signalosomes. It provides a preclinical model for IgG4-related disease, a human autoimmune and inflammatory condition.

## Graphical Abstract

**Figure:**
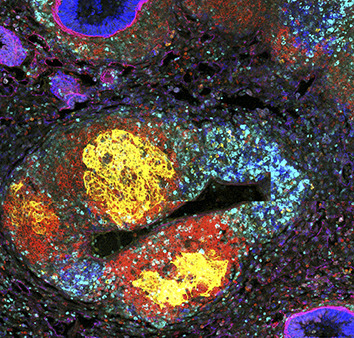

## Introduction

TCR signaling is essential for the development and function of T cells, and its malfunction has pathological consequence (Tangye et al., 2021). The membrane-proximal TCR signal-transduction apparatus can be broken down into an antigen-recognition and triggering module made of the TCR–CD3 complex and the LCK and ZAP-70 protein tyrosine kinases, and into a signal diversification module based on the LAT transmembrane adaptor (Malissen and Bongrand, 2015). Upon ZAP-70–mediated phosphorylation, several tyrosine residues of LAT cooperatively bind cytosolic signaling and adaptor molecules to give rise to a protein signaling complex known as the LAT signalosome (Balagopalan et al., 2010; Lo and Weiss, 2021; Nicolas et al., 2022; Wada et al., 2022). It controls cytoskeletal dynamics, metabolism, transcription, and translation, and is responsible for most of the early and late responses resulting from TCR engagement (Mori et al., 2021). The ability of the LAT signalosome to activate the NFAT and RAS–MAPK signaling pathways via phospholipase PLC-γ1 accounts for the transcriptional response induced by the TCR (Ashouri et al., 2021; Balagopalan et al., 2010; Lo and Weiss, 2021; Mori et al., 2021).

Small numbers of CD4^+^ T cells expressing overtly autoreactive TCR develop in the hypocellular thymus of mice homozygous for a LAT mutation that corresponds to a replacement of the tyrosine found at position 136 with phenylalanine and abrogates the interaction of LAT with PLC-γ1 (denoted as *Lat*^Y136F^ mice; Aguado et al., 2002; Sommers et al., 2002). After reaching secondary lymphoid organs (SLO) and engaging their autoreactive TCR, *Lat*^Y136F^ CD4^+^ T cells convert into activated T cells that give rise to a polyclonal lymphoproliferative disorder that reaches a plateau 6–8 wk after birth (Aguado et al., 2002; Sommers et al., 2005). Expanding *Lat*^Y136F^ CD4^+^ T cells adopt a type 2 polarization and trigger a massive polyclonal B cell activation resulting 6 wk after birth in serum IgG1 and IgE concentrations elevated 200 and 10,000 times, respectively, as compared with age-matched wild-type (WT) mice (Aguado et al., 2002; Genton et al., 2006). Therefore, a distinctive pathological condition, called “defective LAT signalosome pathology” (DLSP), ensues in mouse when naive CD4^+^ T cells expressing defective LAT signalosomes are activated via their TCR (Mingueneau et al., 2009).

2-mo-old *Lat*^Y136F^ mice develop systemic autoimmunity as documented by the presence of autoantibodies directed to DNA and kidney and salivary gland autoantigens (Genton et al., 2006; Sommers et al., 2002). Dense infiltrates made of CD4^+^ T cells and IgG1-producing plasma cells (PC) are found in the lung, liver, salivary glands, pancreas, kidney, and dura mater of *Lat*^Y136F^ mice with occasional fibrosis and eosinophilia (Aguado et al., 2002; Cui et al., 2019; Genton et al., 2006; Honda et al., 2021; Waseda et al., 2021; Yamada et al., 2018). In view of this spectrum of histopathological manifestations and considering that mouse IgG1 constitutes the homologue of human IgG4, it has been suggested that the *Lat*^Y136F^ DLSP constitutes a mouse model of human IgG4-related disease (IgG4-RD; Yamada et al., 2018), a fibro-inflammatory condition originally characterized by high levels of IgG4. The multiorgan tumor-like masses characteristic of IgG4-RD involve T follicular helper T cells (Tfh), CD4^+^ cytotoxic T lymphocytes (CTL), and IgG4^+^ PC, and lead to irreversible tissue damage (Chen et al., 2019; Katz and Stone, 2022; Maehara et al., 2023; Perugino and Stone, 2020). Moreover, IgE is often increased in the serum of IgG4-RD patients (Della Torre et al., 2014).

The pathogenic CD4^+^ T cells developing in *Lat*^Y136F^ mice have been solely analyzed in bulk, precluding to determine whether the *Lat*^Y136F^ DLSP constitutes at single-cell resolution an authentic preclinical model of human IgG4-RD. Here, using single-cell transcriptomics and functional genomics, we assessed the heterogeneity and function of the pathogenic T and B cells expanding in the spleen and lung of *Lat*^Y136F^ mice over 9 wk after birth. It demonstrated that the *Lat*^Y136F^ DLSP qualifies at the single-cell level as a preclinical model of IgG4-RD. Moreover, by visualizing the earliest stages of the *Lat*^Y136F^ DLSP, we elucidated its causative molecular and cellular events, demonstrating how *Lat*^Y136F^ CD4^+^ T cells trigger an early onset autoimmune inflammation.

## Results

### The CD4^+^ T cells expanding in *Lat*^Y136F^ spleen are heterogeneous

**Figure 1.**
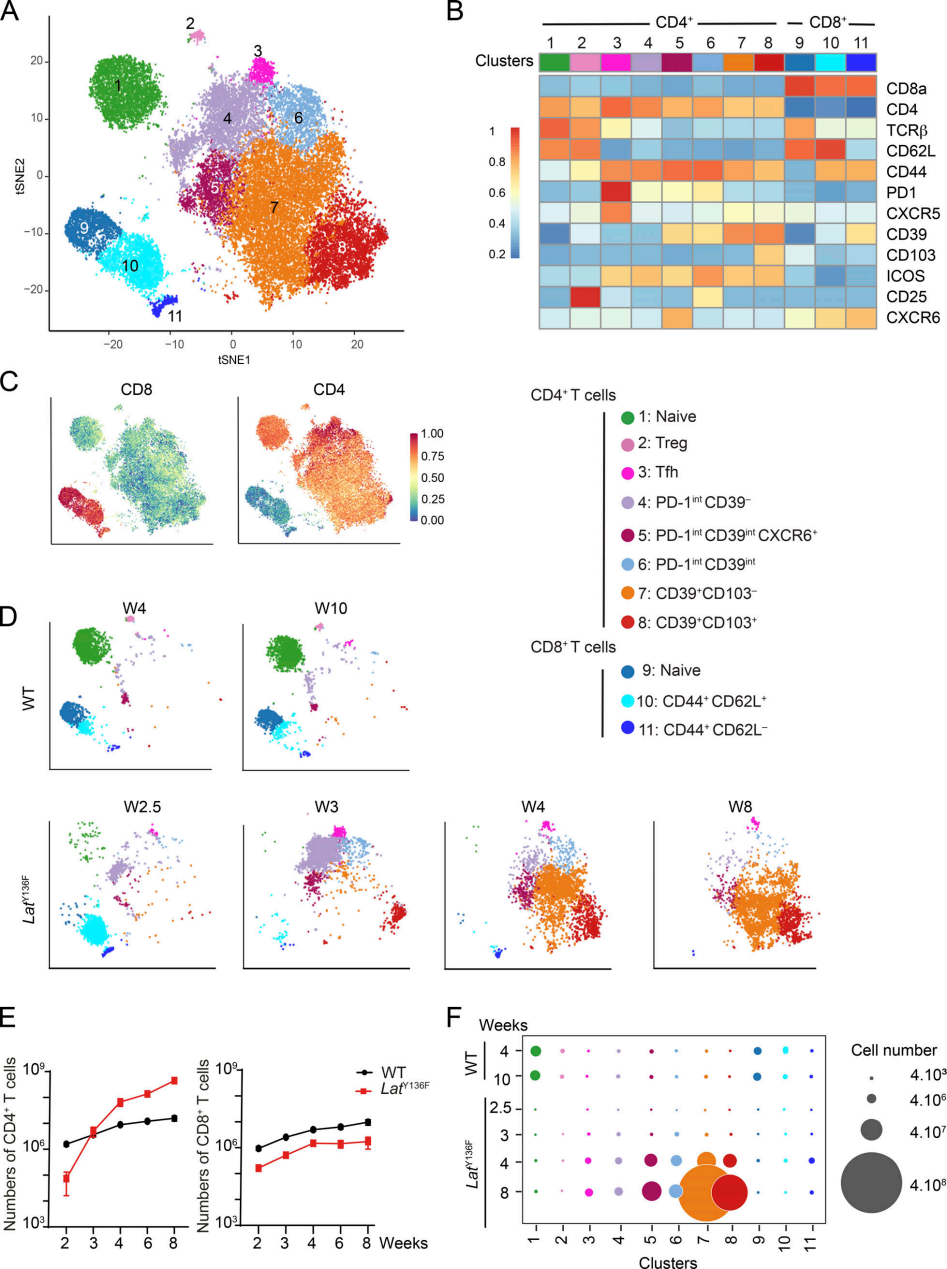
**Flow-cytometry analysis of the *Lat***^**Y136F**^ **DLSP onset. (A)** A multiplex antibody panel was used to analyze by flow cytometry the T cells found in the spleen of 2.5-, 3-, 4-, and 8-wk-old *Lat*^Y136F^ mice and of 4- and 10-wk-old WT mice. The six datasets were merged and subjected to tSNE and unsupervised clustering. The composite two-dimensional tSNE scatterplot revealed the presence of 11 T cell clusters that are color-coded (see key). **(B)** Heatmap showing the intensity of expression of the specified T cell surface markers within each of the CD4^+^ and CD8^+^ T cell clusters identified in A. **(C)** Expression of CD4 and CD8 across the tSNE scatterplot. **(D)** Deconvolution of the composite tSNE scatterplot shown in A into its WT components at 4 and 10 wk (W) after birth, and its *Lat*^Y136F^ components at 2.5, 3, 4, and 8 wk after birth. **(E)** Absolute numbers of CD4^+^ and CD8^+^ T cells found in the spleen of WT and *Lat*^Y136F^ mice at the specified ages. In E and F, at least two experiments were performed involving two to four mice per genotype, and the mean and SEM are shown. **(F)** Numbers of WT and *Lat*^Y136F^ splenic T cells found in the cell clusters (see A) specified on the x axis at the ages indicated on the y axis. The dot size is commensurate to the number of cells present in the specified T cell cluster (see key).

To determine the heterogeneity of the T cells responsible for the *Lat*^Y136F^ DLSP, we developed a multiplex antibody panel probing 14 T cell surface markers. Considering that the *Lat*^Y136F^ DLSP is fully established between 6 and 8 wk after birth (Archambaud et al., 2009), a period denoted here as the “end-state,” we applied such a panel to T cells from the spleen of 2.5-, 3-, 4-, and 8-wk-old *Lat*^Y136F^ mice and of 4- and 10-wk-old WT mice. The six resulting flow cytometric datasets were merged and subjected to dimensionality reduction using t-stochastic neighbor embedding (tSNE), and to unsupervised clustering using the PhenoGraph algorithm (see Materials and methods). It revealed the presence of 11 cell clusters (Fig. 1, A and B).

**Figure S1.**
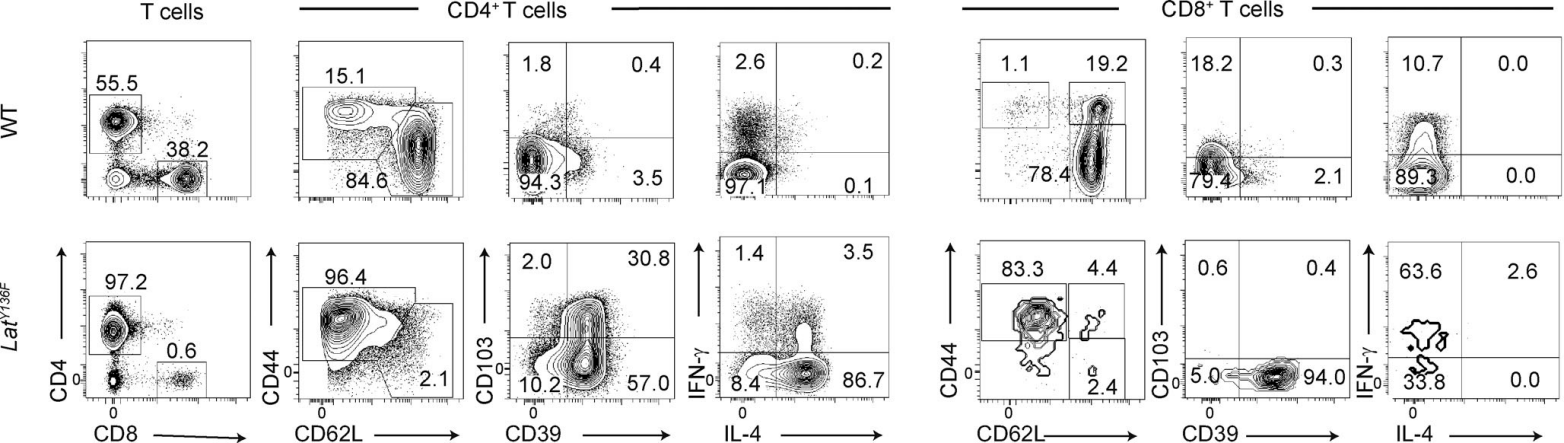
**Flow cytometry analysis of the end-state T cell populations found in *Lat***^**Y136F**^ **spleens.** CD4^+^ and CD8^+^ T cells isolated from the spleen of 8-wk-old WT (top) or *Lat*^Y136F^ (bottom) mice were analyzed by flow cytometry for the specified markers. Also shown is their production of IL-4 and IFN-γ after stimulation with PMA and ionomycin for 4 h in the presence of monensin. The percentage of cells found within each of the specified gates is indicated. Data are representative of at least three independent experiments, each involving three to six mice.

Deconvolution of the composite tSNE plot into its WT and *Lat*^Y136F^ components showed that WT CD4^+^ and CD8^+^ T cells had a predominant CD44^−^CD62L^+^ naive phenotype (clusters 1 and 9; Fig. 1, B–D, and Fig. S1). In contrast, 2.5 wk after birth, most naive *Lat*^Y136F^ CD8^+^ T cells (cluster 9) had already converted into CD44^+^CD62L^+^ (cluster 10) and CD44^+^CD62L^−^ (cluster 11) activated CD8^+^ T cells. Likewise, naive *Lat*^Y136F^ CD4^+^ T cells (cluster 1) converted between 2.5 and 3 wk after birth into CD44^+^CD62L^−^ activated CD4^+^ T cells, corresponding to CXCR5^+^PD-1^+^ICOS^+^ Tfh cells (cluster 3) and to T cells expressing intermediate levels of PD-1 (clusters 4, 5, and 6; Fig. 1, B–D). The size of the activated CD8^+^ T cell clusters plateaued around 4 wk after birth, and their percentages among T cells started decreasing when CD39^+^ CD4^+^ T cells that lacked (cluster 7) or expressed (cluster 8) CD103 appeared around 4 wk after birth and started expanding (Fig. 1, D–F). Consistent with their expected type 2 polarization (Aguado et al., 2002), 90% of end-state *Lat*^Y136F^ CD4^+^ T cells produced IL-4 after in vitro activation with PMA and ionomycin (Fig. S1). Therefore, despite the 10-fold expansion manifested by *Lat*^Y136F^ CD8^+^ T cells between 2 and 8 wk after birth, they were rapidly outnumbered by *Lat*^Y136F^ CD4^+^ T cells which expanded 6,000-fold over the same period (Fig. 1, E and F).

### Single-cell RNA sequencing (scRNAseq) analysis of *Lat*^Y136F^ T cells during DLSP development

**Figure 2.**
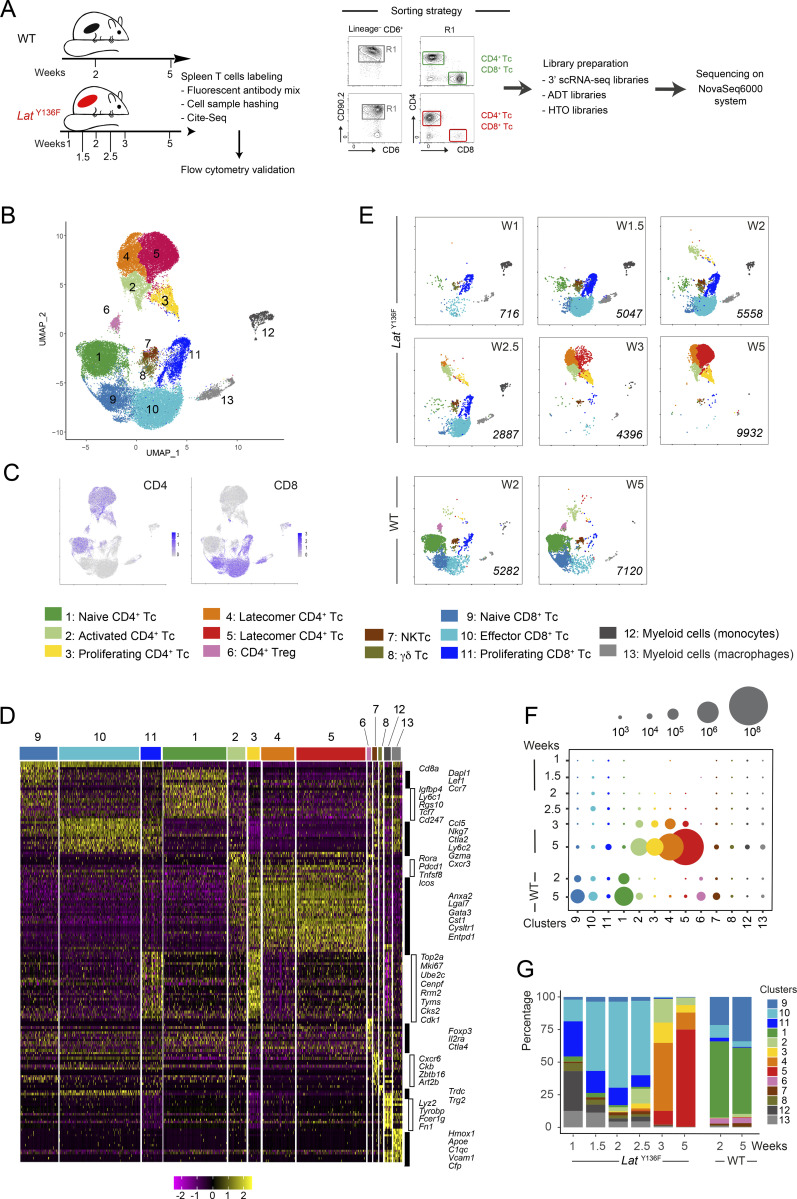
**scRNAseq analysis of *Lat***^**Y136F**^ **CD4**^**+**^ **and CD8**^**+**^ **T cells from 1 to 5 wk after birth. (A)** Workflow schematic for isolating αβ T cells from WT and *Lat*^Y136F^ spleens and processing them for scRNAseq analysis. Due to the low levels of TCR–CD3 expressed at their surface, CD4^+^ and CD8^+^ *Lat*^Y136F^ T cells (Tc) were sorted using a combination of CD90.2 and CD6 antibodies rather than via CD3. **(B)** Unsupervised clustering performed on a UMAP representation corresponding to the eight sorted samples and calculated based on the 30 first principal components of a PCA that used the 2,000 most variable genes as input. Each dot corresponds to one individual cell. 13 clusters were identified and color coded (see key). Two distinct clusters (4 and 5) of latecomer CD4^+^ T cells were identified. **(C)** Expression of CD4 and CD8 across the UMAP representation. **(D)** Heatmap with unsupervised hierarchical clustering showing the expression of the top 158 DEGs. Cells are grouped according to the clusters defined in A as indicated at the top of the heatmap, and each row corresponds to one DEG. A selection of genes specifically expressed within each of the 13 clusters is shown on the right side. **(E)** Deconvolution of the composite UMAP plot shown in B into its WT component at 2 and 5 wk (W) after birth, and its *Lat*^Y136F^ components at 1, 1.5, 2, 2.5, 3, and 5 wk after birth. The number of single cells from which mRNA-seq data were successfully recorded is indicated in italics for each condition. Based on diagnostic transcripts (Fig. 2 D and Data S1), clusters 7, 8, 12, and 13 corresponded to natural killer T cells (NKTc), γδ T cells, and myeloid cells. They contaminated the minute numbers of conventional αβ T cells present in the spleen at the earliest time points and were not analyzed further. **(F)** Numbers of WT and *Lat*^Y136F^ T cells found in the cell clusters (see key) specified on the x axis and at the ages indicated on the y axis. The dot size is commensurate to the number of cells present in the specified T cell clusters. **(G)** Contribution (%) of each of the 13 identified T cell clusters to the cell populations sorted from WT and *Lat*^Y136F^ spleen at the ages specified on the x axis. In F and G, spleens corresponding to seven 2-wk-old and two 5-wk-old WT mice, nine 1-wk-old, 11 1.5-wk-old, six 2-wk-old, five 2.5-wk-old, three 3-wk-old, and one 5-wk-old *Lat*^Y136F^ mice were collected. Spleen cells corresponding to each condition were pooled. Prior to subjecting them to T cell enrichment, cell sorting, and subsequent scRNAseq analysis (Fig. 2 A and Materials and methods), an aliquot of each spleen cell pool was analyzed by flow cytometry for the expression of CD4 and CD8 and used to determine the absolute numbers of CD4^+^ and CD8^+^ T cells present per spleen for each of the eight conditions. Those numbers were combined with the percentages of cells corresponding to each of the 13 clusters defined in the UMAP representation shown in B, allowing the calculation of the cell numbers and percentages shown in F and G.

**Figure S2.**
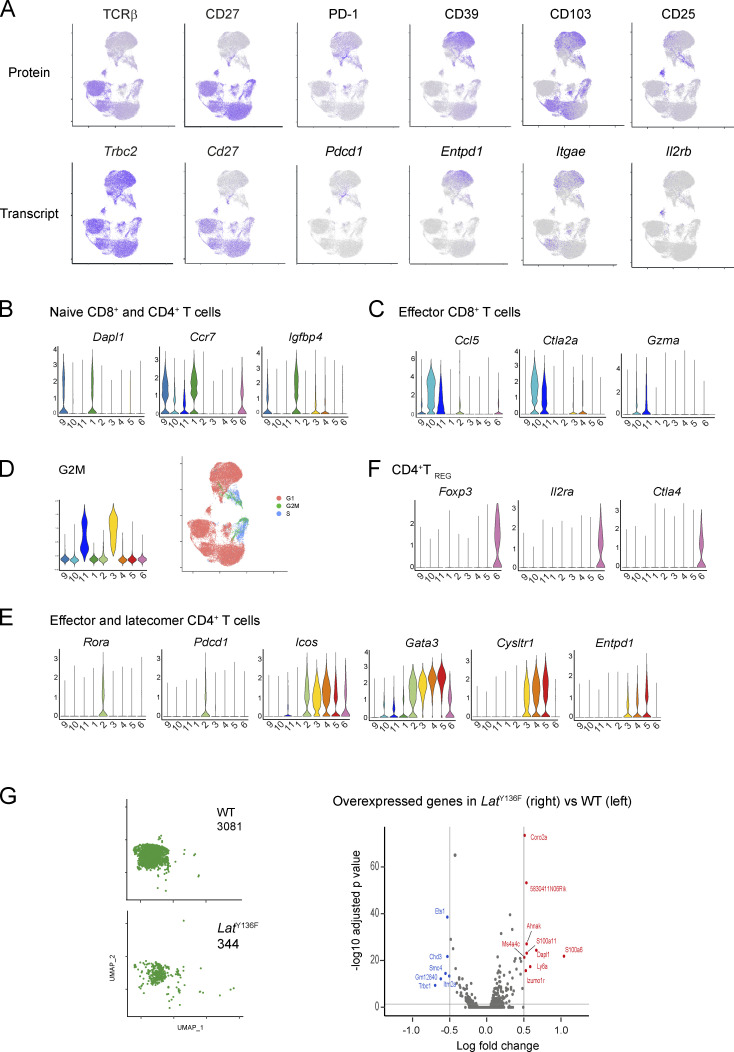
**Characterization of the CD4**^**+**^ **and CD8**^**+**^ **cell clusters described in** Fig. 2**. (A)** UMAP representation illustrating the protein (top) and transcript (bottom) levels of the specified molecules. **(B)** Violin plots of diagnostic genes for naive CD4^+^ (cluster 1) and CD8^+^ (cluster 9) T cells. **(C)** Violin plots of diagnostic genes for effector CD8^+^ (clusters 10 and 11) T cells. **(D)** Left: Violin plots showing the expression of cell cycle genes as determined by Seurat analysis. Right: UMAP representation of the expression of genes corresponding to the G1 (red), S (blue), and G2/M (green) phases of the cell cycle. **(E)** Violin plots of diagnostic genes for effector and latecomer CD4^+^ T cells (clusters 2–5). **(F)** Violin plots of diagnostic genes for Treg cells (cluster 6). **(G)** Left: UMAP of naive CD4^+^ cells of WT and *Lat*^Y136F^ spleen. Right: Volcano plot showing DEGs between WT and *Lat*^Y136F^ naïve CD4^+^ cells. Upregulated and downregulated DEG are shown in red and blue, respectively.

To define the transcriptome of the CD4^+^ and CD8^+^ T cell subsets associated with the onset of the *Lat*^Y136F^ DLSP, we performed scRNAseq analysis on CD4^+^ and CD8^+^ T cells isolated from *Lat*^Y136F^ spleens at 1, 1.5, 2, 2.5, 3, and 5 wk after birth, and from WT spleens at 2 and 5 wk after birth (Fig. 2 A). By focusing on *Lat*^Y136F^ mice up to 5 wk after birth, we prevented the scRNAseq datasets to be obliterated by the massively expanding CD4^+^ cells corresponding to flow-cytometry clusters 7 and 8 (Fig. 1 F). To align the clusters defined by flow cytometry and scRNAseq analyses (Fig. 2 A), we used a cellular indexing of transcriptomes and epitopes by sequencing (CITE-seq) approach (Stoeckius et al., 2017). Accordingly, purified CD4^+^ and CD8^+^ T cells were labeled before sorting with a mix of oligonucleotide-tagged antibodies directed at CD27, CD25, CD39, TCRβ, PD-1, and CD103 (Fig. S2 A). Cell hashing with barcoded antibodies was also used for multiplexing and exclusion of multiplets (Stoeckius et al., 2018). Most of the sequenced T cells passed the quality controls (see Materials and methods), and we detected a mean of 2,241 genes per cell and a total of 20,967 genes. A uniform manifold approximation and projection (UMAP) representation encompassing all the sorted samples was calculated based on the first 30 principal components of a principal component analysis (PCA) that used an input corresponding to the 2,000 most variable genes. Unsupervised clustering revealed the presence of 13 cell clusters (Fig. 2 B and Data S1).

### Expansion of *Lat*^Y136F^ CD8^+^ T cells preceded that of *Lat*^Y136F^ CD4^+^ T cells

CD8^+^ T cells were distributed among clusters 9, 10, and 11 (Fig. 2, B and C). Based on diagnostic transcripts (Fig. 2 D and Data S1), cluster 9 corresponded to naive CD8^+^ T cells and was well represented in the WT samples but poorly represented in *Lat*^Y136F^ samples (Fig. 2, C and E). Clusters 10 and 11 were present in both WT and *Lat*^Y136F^ samples. Cluster 10 expressed genes characteristic of CD8^+^ effector T cells whereas cluster 11 corresponded to their proliferating counterpart (Fig. 2 D and Fig. S2, B–D). 1 wk after birth, most of the naive *Lat*^Y136F^ CD8^+^ T cells had already converted into resting and proliferating effector CD8^+^ T cells (Fig. 2 E). As a result, *Lat*^Y136F^ CD8^+^ effector T cells represented ∼90% of the T cells found in the spleen of 2.5-wk-old *Lat*^Y136F^ mice (Fig. 2, F and G). Although *Lat*^Y136F^ CD8^+^ effector T cells steadily expanded over the following weeks, their frequency among T cells markedly decreased when *Lat*^Y136F^ CD4^+^ activated T cells (clusters 2–5) started expanding around 3 wk after birth (Fig. 2, E–G), a result consistent with our flow cytometry analysis (Fig. 1).

### *Lat*^Y136F^ and WT naive CD4^+^ T cells expressed similar transcriptomes

The CD4^+^ T cells present in 2- and 5-wk-old WT spleens comprised naive (cluster 1), Foxp3^+^ regulatory T (Treg; cluster 6) cells, and a few activated cells (Fig. 2, E–G; and Fig. S2, B, F and E). At 1 and 1.5 wk after birth, most of the *Lat*^Y136F^ CD4^+^ T cells corresponded to naive cells (cluster 1; Fig. 2, E–G). To assess the transcriptomic similarity existing between the naive CD4^+^ T cells found in WT and *Lat*^Y136F^ spleens, we merged those found in all the *Lat*^Y136F^ samples (representing 344 cells) and compared them with those from 2-wk-old WT spleen (representing 3,081 cells). They shared an αβTCR^+^CD62L^+^CD44^low^CD27^+^ phenotype and only differed by the expression of 15 genes, of which 14 showed less than twofold difference in expression, and the biological relevance of which remains to be determined (Fig. S2 G). Therefore, prior to engaging their autoreactive TCR and converting into activated T cells, *Lat*^Y136F^ naive CD4^+^ T cells expressed a transcriptome almost similar to that of WT naive CD4^+^ T cells.

### Single-cell transcriptomics analysis of *Lat*^Y136F^ CD4^+^ T cell diversification

**Figure 3.**
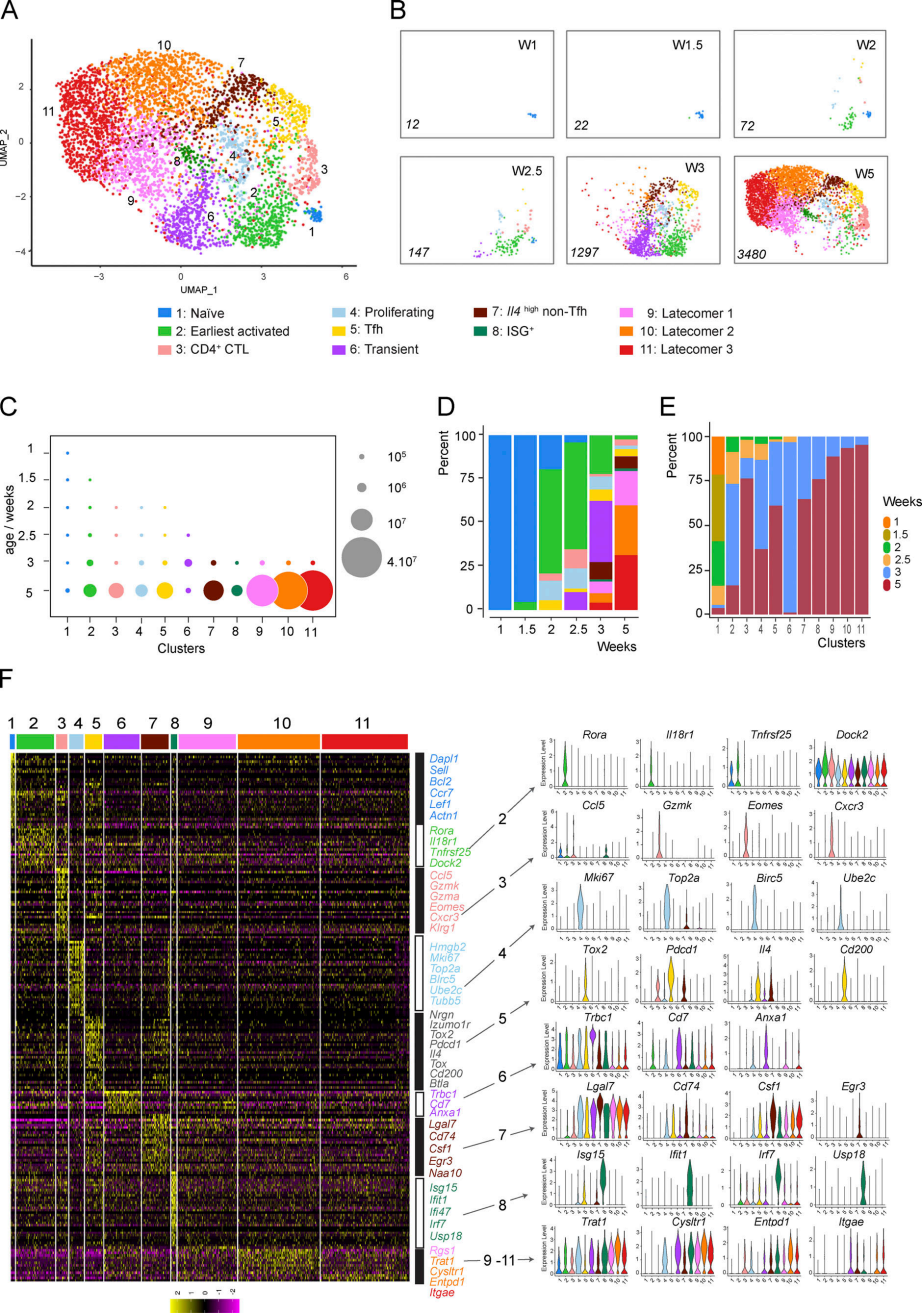
**scRNAseq analysis of *Lat***^**Y136F**^ **CD4**^**+**^ **T cells across six time points straddling DLSP development. (A)** Composite UMAP representation of splenic *Lat*^Y136F^ CD4^+^ T cells corresponding to the six analyzed time points and calculated based on the first 30 principal components of a PCA. 11 clusters were identified, numbered according to their order of appearance, and color-coded (see key). **(B)** Deconvolution of the composite UMAP representation shown in A into its components corresponding to 1, 1.5, 2, 2.5, 3, and 5 wk (W) after birth. The number of single cells from which mRNA-seq data were successfully recorded is indicated in italics for each condition. **(C)** Numbers of splenic *Lat*^Y136F^ CD4^+^ T cells found in the cell clusters specified on the x axis at the ages indicated on the y axis. The dot size is commensurate to the number of cells present in the specified cluster (see key). **(D)** Contribution (%) of each of the 11 identified CD4^+^ T cell clusters (see key) to the cell populations sorted from WT and *Lat*^Y136F^ spleen at the ages specified on the x axis. **(E)** Contribution (%) of each of the 11 *Lat*^Y136F^ CD4^+^ T cell clusters at the six analyzed time points. For C, D, and E, see description in Fig. 2, F and G, of the mode of calculation of the cell numbers and percentages. **(F)** Heatmap with unsupervised hierarchical clustering showing the expression of the top 212 DEGs. Cells are grouped according to the clusters defined in A as indicated at the top of the heatmap, whereas each row corresponds to one DEG. A selection of genes specifically expressed within each of the 11 clusters is shown on the right side together with select violin plots.

We next determined whether the *Lat*^Y136F^ CD4^+^ T cell diversification and functional specification that started 2 wk after birth resembled those previously described in physiological and pathological conditions. 11 *Lat*^Y136F^ CD4^+^ T cell clusters were identified using unsupervised hierarchical clustering of a UMAP representation of 5,030 *Lat*^Y136F^ CD4^+^ T cells corresponding to all the analyzed time points (Fig. 3, A and B). Each cluster received a specific number according to its order of developmental appearance. The numbers of cells corresponding to nine of the 10 activated cell clusters increased over the analyzed period, whereas cluster 6 cells diminished 3 wk after birth and are thus denoted as “transient” CD4^+^ T cells (Fig. 3, C–E).

**Figure S3.**
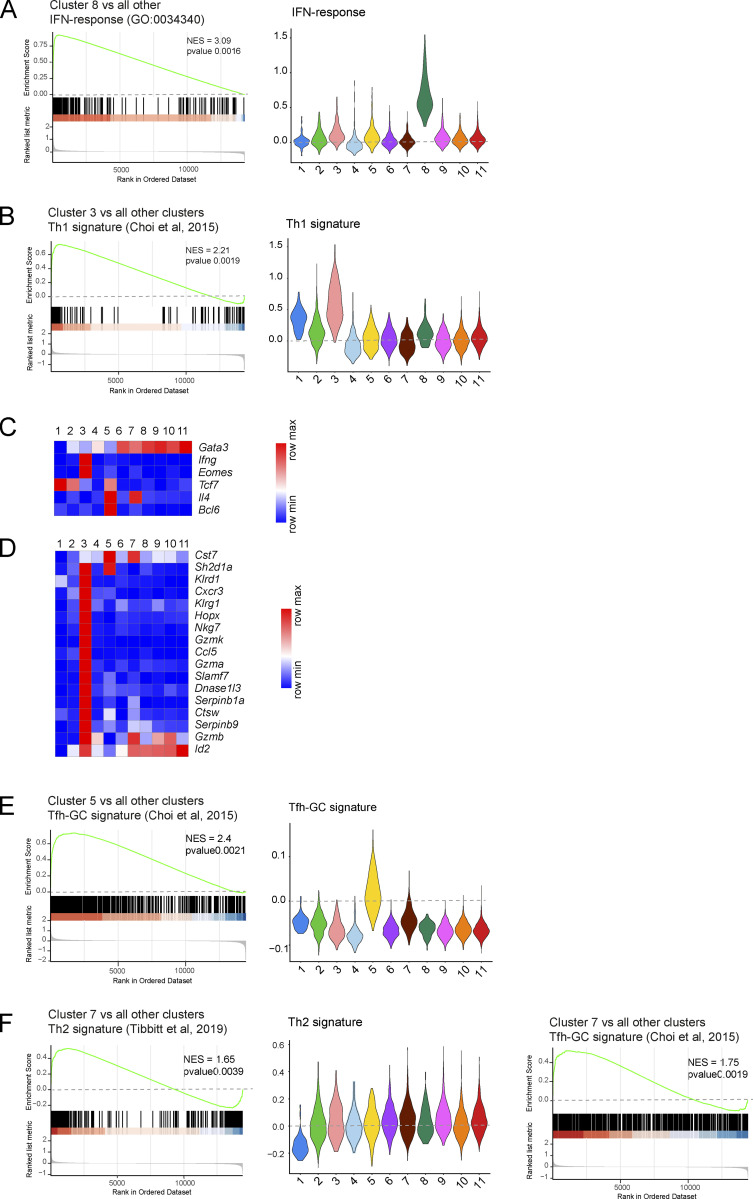
**Gene signatures corresponding to clusters 3, 5, 7, and 8 identified in scRNAseq analysis of *Lat***^**Y136F**^ **CD4**^**+**^ **T cells. (A)** Left: GSEA comparing ISG^+^ cells (cluster 8) versus all other cell clusters for genes involved in type I IFN response. Right: Violin plots showing the expression of genes involved in type I IFN response in the clusters specified on the x axis. **(B)** Left: GSEA comparing CD4^+^ CTL (cluster 3) versus all other cell clusters for a Th1 cell gene signature (Tibbitt et al., 2019). Right: Violin plots showing the expression of genes involved in Th1 effector cells in the clusters specified on the x axis. **(C)** Heatmap analysis of the expression of select genes coding for transcription factors and cytokines. The color scale is row-normalized and corresponds to the relative fold change. **(D)** Heatmap analysis of the expression of select DEG of CD4^+^ CTL (Hashimoto et al., 2019) among the 11 clusters of *Lat*^Y136F^ CD4^+^ T cells. The color scale is row-normalized and corresponds to the relative fold change. Gene names are listed on the right and cluster numbers at the top. **(E)** Left: GSEA comparing Tfh cells (cluster 5) versus all other cell clusters for genes highly expressed in GC Tfh cells (Choi et al., 2015). Right: Violin plots showing the expression of genes found to be highly expressed in GC Tfh cells in the clusters specified on the x axis. **(F)** Left: GSEA comparing *Il4*^high^ non-Tfh cells (cluster 7) versus all other cell cluster for a Th2 cell gene signature (Tibbitt et al., 2019). Middle: Violin plots showing the expression of the genes highly expressed in Th2 effector cells in the clusters specified on the x axis. Right: GSEA plot comparing *Il4*^high^ non-Tfh cells (cluster 7) versus all other cells for genes found to be highly expressed in GC Tfh cells. NES, normalized enrichment score.

Genes specifically expressed in each of the 11 clusters were visualized using a heatmap of the top 212 differentially expressed genes (DEG) and violin plot representations (Fig. 3 F and Datas S2 and S3). Cluster 1 corresponded to CD44^low^CD62L^high^ naive CD4^+^ T cells, whereas cluster 2 cells had upregulated *Cd5*, *Cd6*, and *Cd44,* downregulated *Cd27* (coding for TNFRSF7), and *Sell* (coding for CD62L; Fig. 3 F). Therefore, cluster 2 cells corresponded to the first activated CD4^+^ T cells to appear in the spleen of *Lat*^Y136F^ mice and were denoted as “earliest activated CD4^+^ T cells” (Fig. 3, B–E). All the nine remaining clusters that developed subsequently to cluster 2 shared with it a CD44^high^CD62L^low^ activated phenotype. During antigen-driven, physiological type 2 differentiation in SLO, naive CD4^+^ T cells give rise to Tfh cells, which constitutively express high levels of *Il4* transcripts and produce IL-4 proteins, and to non-Tfh cells that constitutively express low levels of *Il4* transcripts and did not express IL-4 protein unless further activated in nonlymphoid tissues (Mohrs et al., 2005; Reinhardt et al., 2009). Seven out of the 10 *Lat*^Y136F^ CD4^+^ cell clusters with an activated phenotype constitutively expressed low levels of *Il4* transcripts, two expressed high levels of *Il4* transcripts, and one expressed *Ifng* transcripts in lieu of *Il4* transcripts (Data S3). The earliest activated CD4^+^ T cells (cluster 2) belonged to those constitutively expressing low levels of *Il4* transcripts. They specifically expressed *Rora*, *Il18r1*, and *Tnfrsf25* (Fig. 3 F), a finding consistent with the view that expression of the *Rora*-encoded nuclear receptor controls genes important for CD4^+^ T cell activation under type 2 polarization condition (Haim-Vilmovsky et al., 2021). Cluster 4 cells also expressed low levels of constitutive *Il4* transcripts, and based on their cell proliferation signature, we denoted them as “proliferating CD4^+^ T cells” (Fig. 3 F and Data S3). The transient CD4^+^ T cells found in cluster 6 expressed low levels of constitutive *Il4* transcripts and were characterized by the expression of *Cd7*. In contrast to the 10 other clusters that predominantly used *Trcb2* transcripts to produce TCRβ chains, cluster 6 cells mostly used *Trbc1* transcripts (Fig. 3 F). Cells in cluster 8 expressed low levels of constitutive *Il4* and were the sole among the 11 clusters to express IFN-stimulated genes (ISG; Fig. 3 F), a finding supported by gene set enrichment analysis (GSEA; Fig. S3 A). They are thus denoted as ISG^+^ CD4^+^ T cells.

Clusters 9, 10, and 11 appeared between 4 and 5 wk after birth and then dominated the CD4^+^ T cell population (Fig. 3, B–E). Those cells that are denoted as “latecomer CD4^+^ T cells” and can be aligned with flow cytometry–defined clusters 7 and 8 based on the expression of *Entpd1* that codes for CD39 (Fig. 1 and Fig. 3 F). Clusters 9, 10, and 11 cells expressed high levels of type 1 cysteinyl leukotriene receptor (*Cysltr1*) transcripts and low levels of constitutive *Il4* transcripts. We failed assigning them to known physiological or pathological CD4^+^ T cell subsets.

### Two *Lat*^Y136F^ CD4^+^ T cell effector clusters match those causative of human IgG4-RD

Cells in cluster 3 were characterized by the expression of *Ccl5*, *Gzmk*, *Gzma*, and *Cxcr3* transcripts (Fig. 3 F). Among the 10 clusters of activated CD4^+^ T cells, cluster 3 was the sole to lack constitutive *Il4* transcripts. Cluster 3 cells uniquely expressed constitutive levels of *Ifng* transcripts and showed similarities with Th1 effector cells (Fig. S3, B and C). Rather than transcribing *Tbx21* that codes for T-bet, a Th1-specific transcription factor that controls *Ifng* gene expression, cluster 3 cells expressed *Eomes* transcripts that code for a T-bet paralog called eomesodermin and capable of mediating T-bet–independent IFN-γ induction in CD8^+^ T cells. Eomesodermin also promotes expression of genes associated with T cell exhaustion (Li et al., 2018), accounting for the presence of *Entpd1*, *Pdcd1*, *Lag3*, *and Klrg1* in cluster 3 cells (Fig. S3 D). The top most DEGs found in cluster 3 cells code for molecules involved in CTL-mediated cytotoxicity (NKG7, GZMK, GZMA, and CTSW), a finding congruent with the view that ectopic expression of eomesodermin in Th2 cells sufficed to trigger expression of CTL-associated genes (Pearce et al., 2003). A comparison of cluster 3 cells with the CD4^+^ CTL present in supercentenarians (Hashimoto et al., 2019) further supported their assignment as CD4^+^ CTL (Fig. S3 D). Akin to the CD4^+^ CTL described in IgG4-RD patients (Della-Torre et al., 2018), the CD4^+^ CTL found in *Lat*^Y136F^ mice expressed the SLAMF7 signaling receptor and its SH2D1A cytosolic adaptor (Data S3).

Clusters 5 and 7 were the sole among the 11 clusters to constitutively express high levels of *Il4* transcripts (Fig. 3 F and Fig. S3 C). Cluster 5 corresponded to Tfh cells based on expression of *Bcl6*, *Cxcr5*, *Pdcd1*, *Il4*, *Icos*, *Tcf7*, *Il21*, and *Tox* (Data S3). Moreover, a GSEA showed that cluster 5 cells were strongly enriched for genes specifically found in germinal center (GC) Tfh cells (Fig. S3 E). Consistent with the view that the BTLA (CD272) co-inhibitory molecule restrains the help delivered to GC B cells by Tfh cells to prevent GC B cell lymphomagenesis (Mintz et al., 2019), cluster 5 was the sole to constitutively express BTLA. A GSEA of the genes differentially expressed by cluster 7 cells showed no obvious Th2 or Tfh cell signature (Fig. S3 F). They lacked both *Bcl6* and *Cxcr5* transcripts (Data S3), and we tentatively denoted them as *Il4*^high^ non-Tfh cells. Interestingly, Tfh cells and, to a lesser degree, *Il4*^high^ non-Tfh cells expressed transcripts coding for neurogranin (*Nrgn*), a protein that regulates the affinity of calmodulin for calcium and is involved in neuronal synaptic plasticity (Data S3). Therefore, the CD4^+^ T cells found in *Lat*^Y136F^ spleen include Tfh cells and CD4^+^ CTL, both of which are thought to be causative of human IgG4-RD.

### Trajectory inference suggests that *Lat*^Y136F^ CD4^+^ cells differentiate along two branches

**Figure S4.**
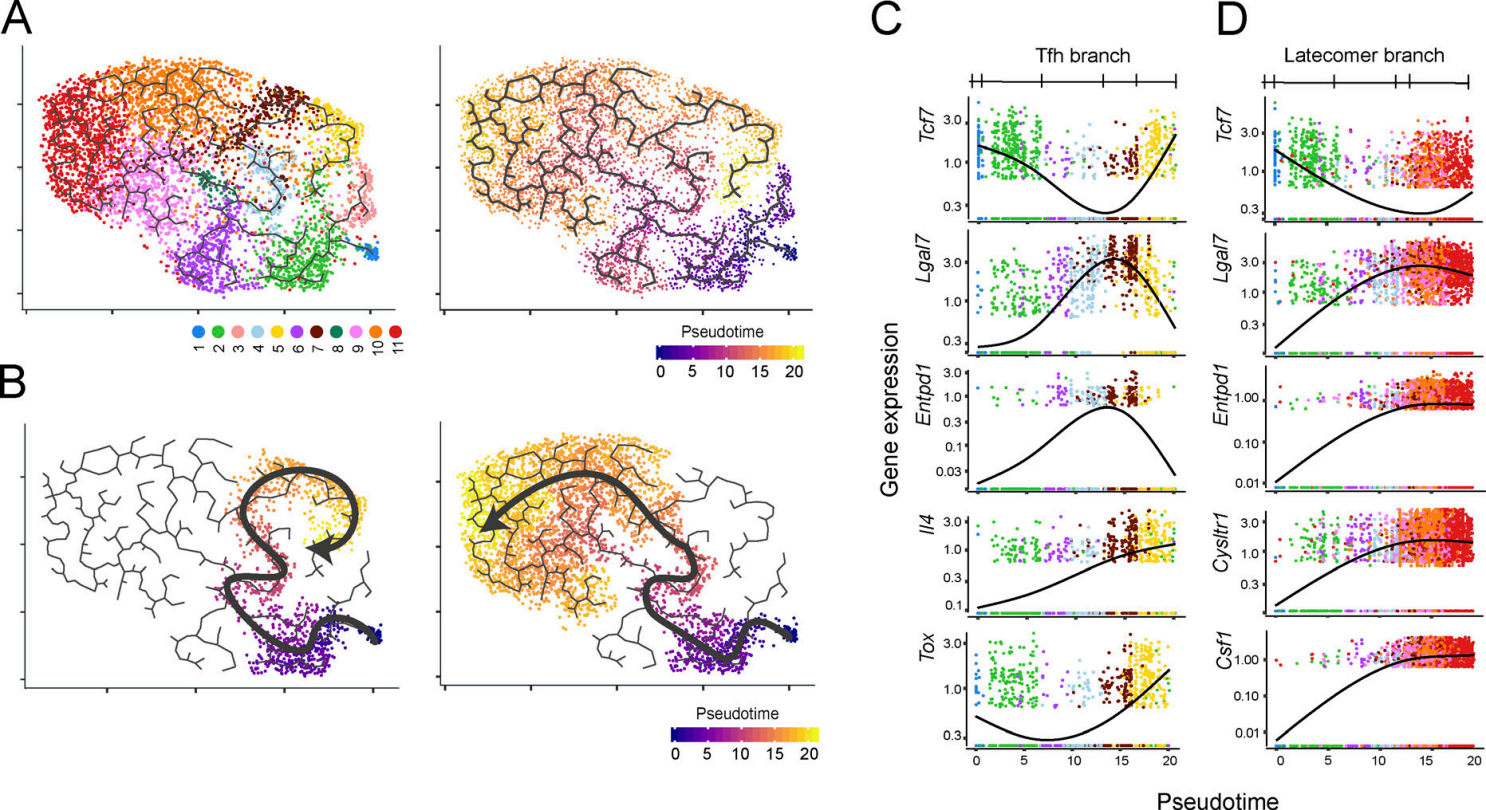
**Modeling the diversification trajectories of *Lat***^**Y136F**^ **CD4**^**+**^ **cells. (A)** UMAP representation of splenic *Lat*^Y136F^ CD4^+^ T cells corresponding to all analyzed time points and colored according to the clusters defined in Fig. 3 A (left) or to pseudotime (right). **(B)** The trajectory leading to Tfh cells (Tfh branch; left) or to latecomer T cells (T latecomer branch; right) are highlighted on the UMAP representation. Both branches are colored according to pseudotime. **(C)** Expression dynamics of selected genes along the Tfh pseudotime trajectory. **(D)** Expression dynamics of selected genes along the T latecomer pseudotime trajectory.

To model the temporal dynamics of the CD4^+^ T cell diversification occurring in *Lat*^Y136F^ mice from birth to 6 wk after birth, we analyzed our scRNAseq dataset using the Monocle 3 trajectory inference method (see Materials and methods). Two distinct trajectories linked the naive CD4^+^ T cell cluster to the most distant clusters corresponding to Tfh cells and to latecomer CD4^+^ T cells (Fig. S4). This inferred order is consistent with the kinetics of appearance of the 11 CD4^+^ T cell clusters and with their levels of expression of *Il4* transcripts and the TCR–CD3 complex (see below). It also fits recent models suggesting that all CD4^+^ T cell responses simultaneously support cell-mediated and humoral immunity via the production of non-Tfh and Tfh cells, respectively (Osum and Jenkins, 2023; Ruterbusch et al., 2020).

### scRNAseq-defined *Lat*^Y136F^ CD4^+^ T cell clusters can be identified via flow cytometry

**Figure S5.**
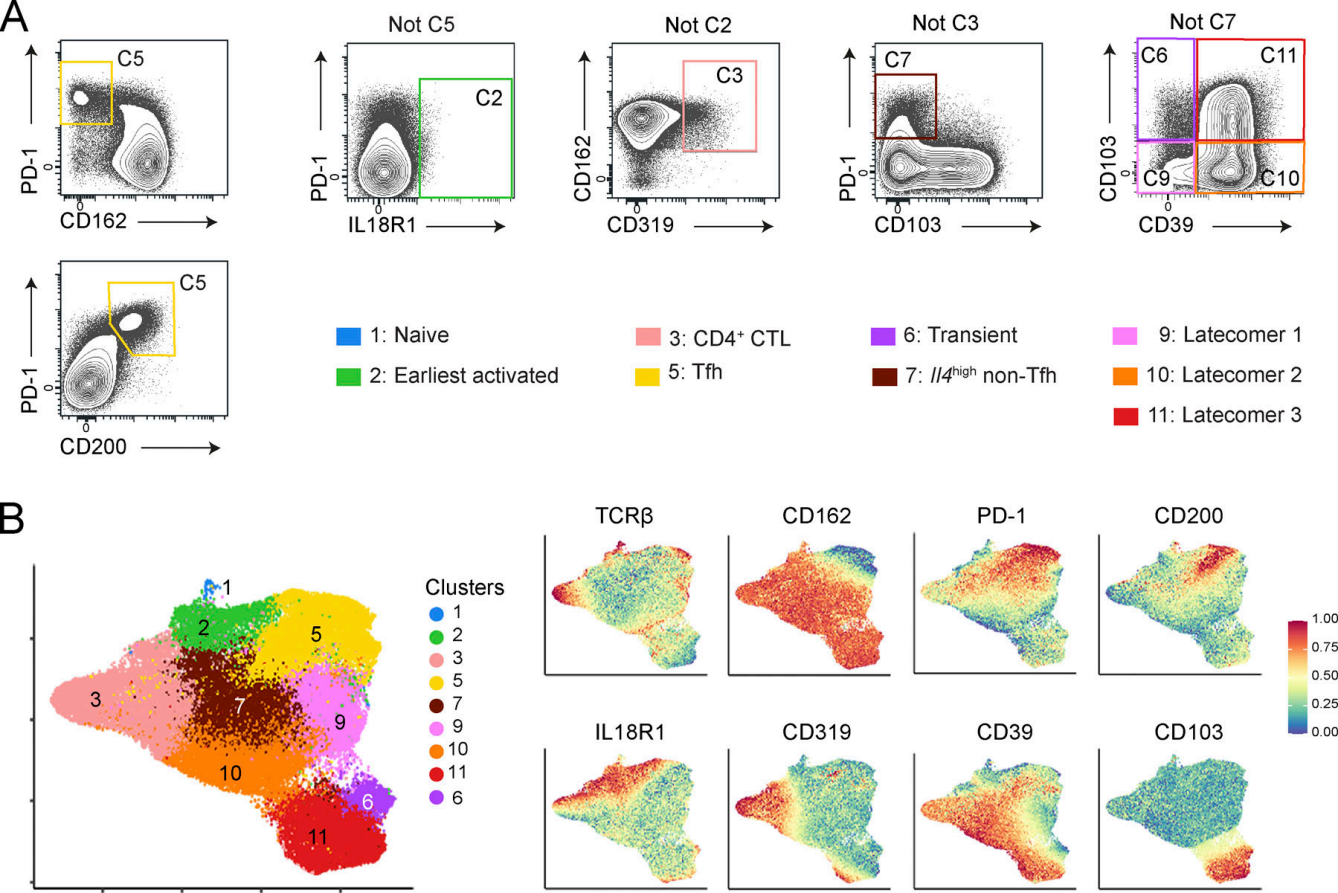
**Flow cytometry gating strategy used to identify the *Lat***^**Y136F**^ **CD4**^**+**^ **T cells subsets originally defined via scRNAseq analysis. (A)** Among single cells from 4-wk-old *Lat*^Y136F^ spleen, CD4^+^ cells were selected for further analysis and examined for the expression of CD103, CD319 (SLAMF7), CD39, CD278 (IL18R1), CD162 (PSGL-1), CD200, and PD-1. The following sequential gating strategy was used. In step 1, cells were analyzed for the expression of PD-1 and CD162 or of PD-1 and CD200, permitting the identification of Tfh cells (C5, yellow gate). In a second step, “not-C5 cells” were analyzed for PD-1 and IL18R1 expression, permitting to define the earliest activated CD4^+^ T cells (C2, green gate). In a third step, “not C5 and not C2 cells” were analyzed for CD162 and CD319, permitting to define CD4^+^ CTL (C3, pink gate). In a fourth step, “not C5, not C2, and not C3 cells” were analyzed for PD-1 and CD103 expression, permitting to define *Il4*^high^ non-Tfh cells (C7, brown gate). In a last step, “not C5, not C2, not C3, and not C7” cells were analyzed for CD103 and CD39 expression to define transient (C6, purple gate) and latecomer CD4^+^ cells (C9, C10, and C11, orange gates). The percentage of cells found within each of the specified gates is indicated. Data are representative of two independent experiments each involving three to four mice. **(B)** Left: Composite UMAP representation of the nine CD4^+^ T cell clusters that were defined by flow cytometry and aligned with those defined by transcriptomics (see key). Right: UMAP representation colored according to the expression level of the specified surface proteins.

**Figure 4.**
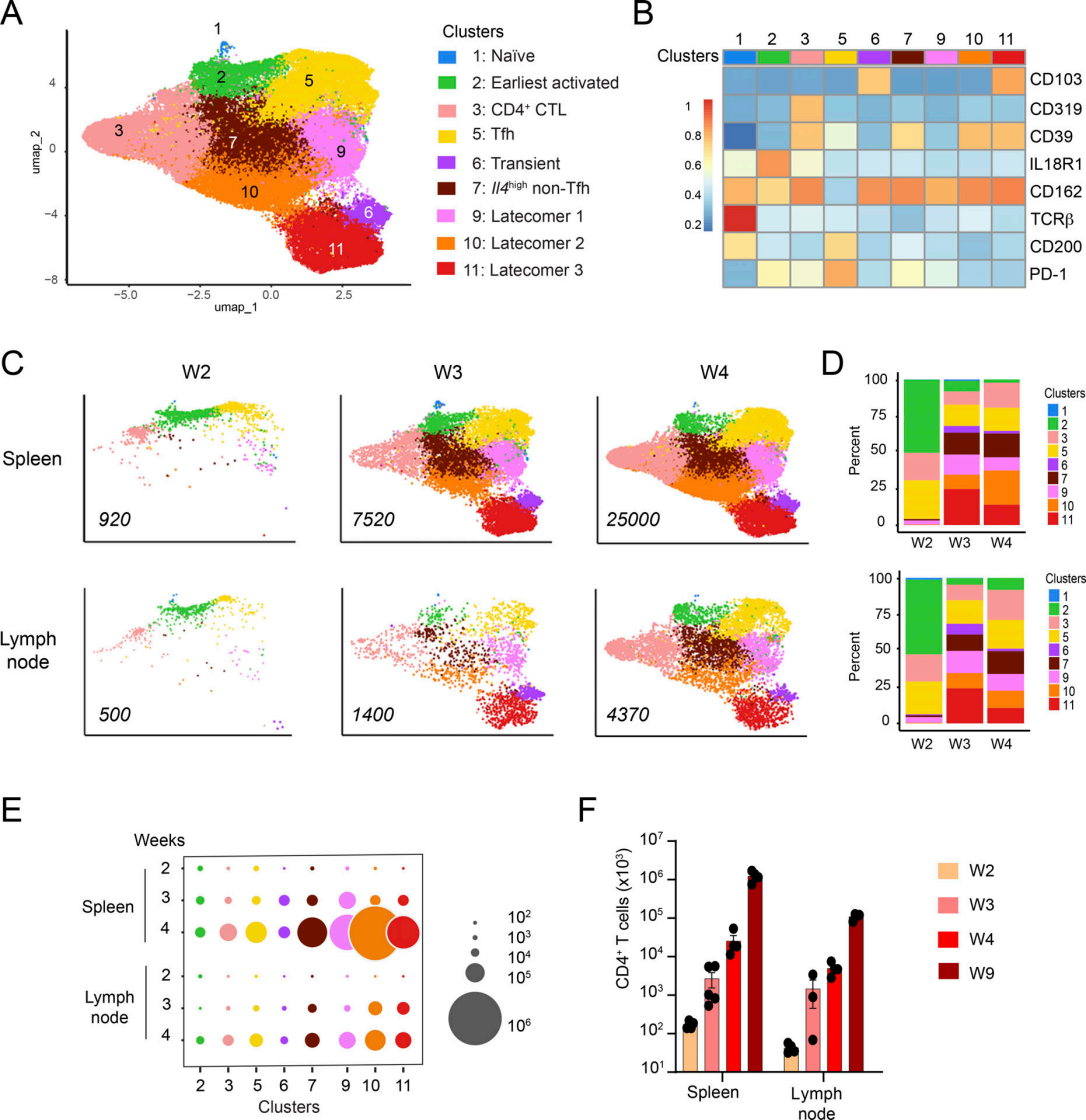
**Flow cytometry–based identification of the CD4**^**+**^ **T cell clusters defined via scRNAseq and analysis of their developmental kinetics in spleen and mLN. (A)** A flow cytometry panel developed on the basis of our scRNAseq dataset was used to analyze the CD4^+^ T cells found in the spleen and mLN of 2-, 3-, and 4-wk-old *Lat*^Y136F^ mice (see Fig. S5). The six flow cytometry datasets were merged and subjected to UMAP and unsupervised clustering. The resulting composite UMAP representation showed that the flow cytometry panel we developed allowed the identification of only 9 out of the 11 CD4^+^ T cell clusters characterized by scRNAseq analysis, clusters 4 and 8 failing to be identified by our flow cytometry panel (see key). **(B)** Heatmap showing the intensity of expression of the specified T cell surface markers within each of the CD4^+^ cell clusters of A (see key). **(C)** Deconvolution of the composite UMAP representation shown in A into its spleen and mLN components at 2, 3, and 4 wk (W) after birth. **(D)** Contribution (%) of each of the nine identified cell clusters to the spleen, and mLN CD4^+^ T cells at the ages specified on the x axis. **(E)** Numbers of CD4^+^ T cells found in the specified cell clusters of the spleen and mLN at the specified ages. The dot size is commensurate to the number of cells present in the specified T cell clusters (see key). Three to four mice were analyzed for each condition, and the mean is represented. In D and E, spleens and mLN isolated from at least four 2-, 3-, and 4-wk-old *Lat*^Y136F^ mice were collected. Prior to separately pooling the spleen and the mLN cell samples and proceeding to dimensionality reduction and unsupervised UMAP clustering (A), an aliquot of each cell pool was analyzed by flow cytometry for the expression of CD4 and used to determine the numbers of CD4^+^ T cells present per spleen or per mLN for each of the three conditions. Those numbers were used together with the percentages of cells corresponding to each of the nine clusters defined in the UMAP representation shown in A to calculate the percentages and cell numbers shown in D and E, respectively. **(F)** Absolute numbers of CD4^+^ T cells found in the spleen and mLN of *Lat*^Y136F^ mice at the specified ages. At least three experiments were performed involving a total of four to five mice per organ and time point, and the mean and SEM are shown.

To further characterize by flow cytometry the *Lat*^Y136F^ CD4^+^ T cell clusters defined via scRNAseq analysis, we mined the scRNAseq dataset for genes coding for cell surface molecules specific for a single or a few defined clusters and for which antibodies were available. It led to a multiplex antibody panel probing eight T cell surface markers (Fig. S5 A). When applied to CD4^+^ T cells isolated from the spleen and mesenteric lymph node (mLN) of 2-, 3-, and 4-wk-old *Lat*^Y136F^ mice, nine out of the 11 cell clusters defined by scRNAseq were readily identified; the two missing clusters corresponded to proliferating and ISG^+^ CD4^+^ T cells (Fig. 4, A and B; and Fig. S5 B).

Deconvolution of the composite UMAP representation corresponding to the six flow cytometry datasets into its spleen and mLN components at 2, 3, and 4 wk after birth confirmed the sequence of CD4^+^ T cell diversification defined for the *Lat*^Y136F^ spleen via scRNAseq and showed that it also occurred in *Lat*^Y136F^ mLN (Fig. 4, C and D). In both spleen and mLN, earliest activated CD4^+^ cells, CD4^+^ CTL, and Tfh cells were readily detected 2 wk after birth. Therefore, the naive CD4^+^ T cells that seed the periphery of *Lat*^Y136F^ neonates disseminated to the spleen and LN where they diversified and expanded at a similar pace, resulting in identical end states (Fig. 4, E and F).

### Activated *Lat*^Y136F^ CD4^+^ T cells express diminished levels of CD3ζ chains

**Figure 5.**
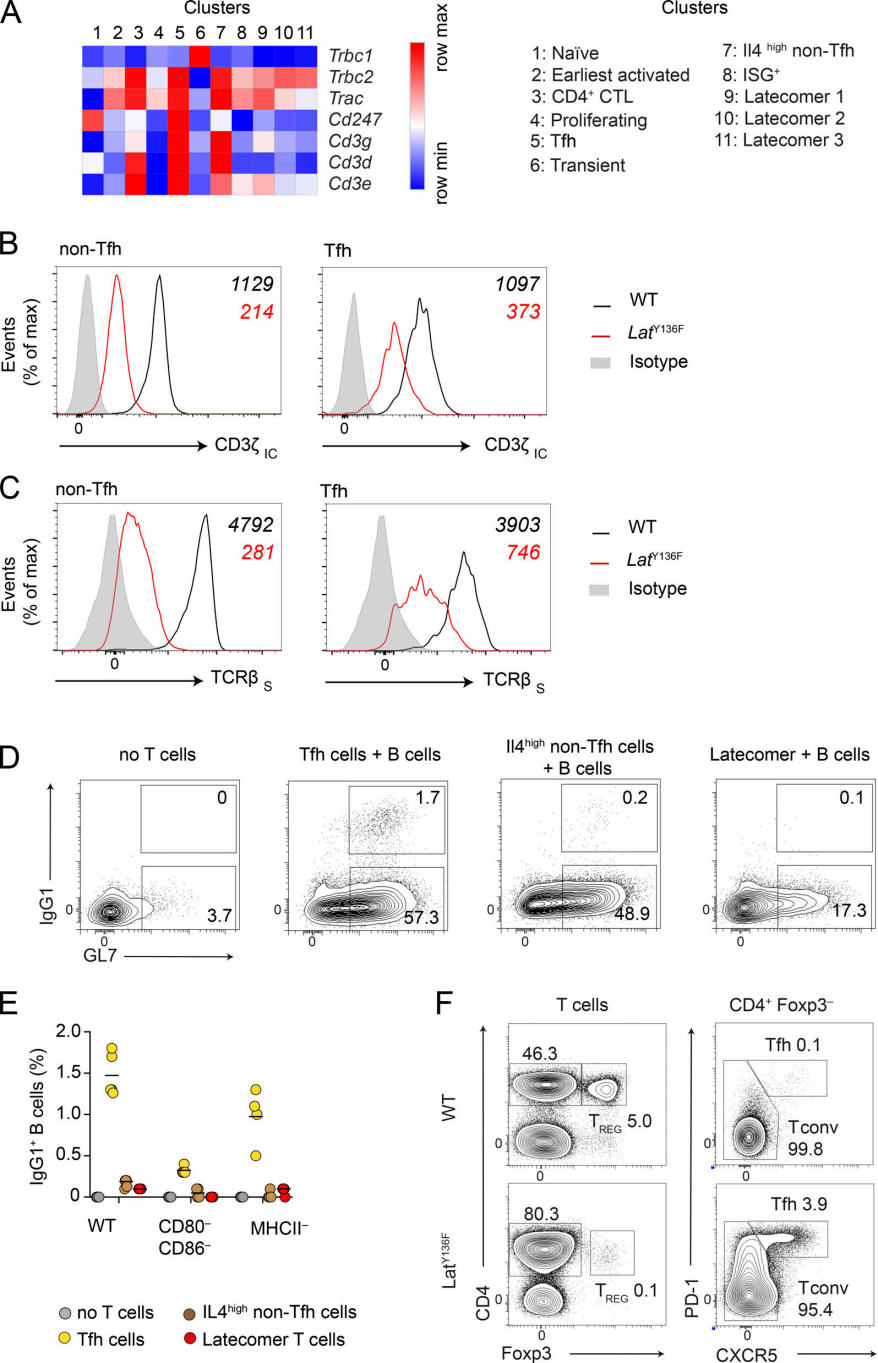
**Characterization of *Lat***^**Y136F**^ **Tfh cells. (A)** Heatmap showing the expression of the transcripts corresponding to the six subunits of the TCR–CD3 complex in each of the 11 CD4^+^ T cell clusters. The color scale is row-normalized and corresponds to the relative fold change. Gene names are listed on the right and cluster numbers at the top (see key). **(B)** CD4^+^ T cells from the spleen of 5-wk-old WT and *Lat*^Y136F^ mice were analyzed by flow cytometry to identify Tfh and non-Tfh cells (in *Lat*^Y136F^ mice, the latter cells comprise both *Il4*^high^ and *Il4*^low ^CD4^+^ T cells and CD4^+ ^CTL; Fig. S5 A), and after a permeabilization step stained with an antibody specific for the intracytoplasmic segment of CD3ζ chains (CD3ζ _IC_). In B and C, background staining was defined using isotype-matched control antibodies (gray-shaded histograms), and numbers correspond to the mean fluorescence intensity of the specified WT (black) and *Lat*^Y136F^ (red) T cells. Data are representative of at least three experiments involving at least two mice per condition. **(C)** The Tfh and non-Tfh cells found among CD4^+^ T cells from the spleen of 5-wk-old WT and *Lat*^Y136F^ mice were analyzed by flow cytometry for surface TCRβ (TCRβ_S_) levels. **(D)** *Lat*^Y136F^ Tfh cells, *Il4*^high^ non-Tfh cells, and latecomer CD4^+^ T cells were sorted according to the strategy shown in Fig. S5 A and cultured with CD19^+^ B cells isolated from WT mice. Plots show the percentage of IgG1^+^ GL7^+^ and IgG1^−^ GL7^+^ GC B cells as determined by flow cytometry after 3 d of culture. **(E)** CD19^+^ B cells isolated from WT, MHCII^−^, or CD80^−^CD86^−^ mice were cultured for 3 d in the absence of *Lat*^Y136F^ CD4^+^ T cells or in the presence of the specified *Lat*^Y136F^ CD4^+^ T cell clusters and quantification of IgG1^+^ B cells performed at the end of the cultures. Each dot corresponds to a mouse, and the mean is shown. Data are representative of two independent experiments involving four mice per condition. **(F)** T cells from WT and *Lat*^Y136F^ spleens were analyzed for expression of Foxp3 and CD4. CD4^+^Foxp3^+^ cells corresponded to Treg cells. Analysis of CD4^+^Foxp3^−^ cells for the expression of CXCR5 and PD-1 permitted to identify CD4^+^Foxp3^−^PD-1^−^CXCR5^−^ conventional T cells (Tconv) and CD4^+^Foxp3^−^PD-1^+^CXCR5^+^ Tfh cells. The percentage of Tfh cells was 39-fold augmented in 8-wk-old *Lat*^Y136F^ spleens. When taking cellularity into account, it corresponded to a 300-fold increase as compared with WT spleens. Note that the percentage of Foxp3^+^ Treg cells in *Lat*^Y136F^ spleen was ∼50-fold reduced as compared with WT spleen. However, when taking cellularity into account, the numbers of Treg cells found in 6-wk-old WT and *Lat*^Y136F^ spleens were almost comparable. Data in F are representative of at least three independent experiments involving three to six mice.

Analysis of the transcripts coding for the subunits of the TCR–CD3 complex (*Trac*, *Trbc1* and *2*, *Cd3g*, *Cd3d*, *Cd3e*, and *Cd247*) showed that those corresponding to *Cd247* and coding for CD3ζ chains were specifically diminished during the conversion of naive *Lat*^Y136F^ CD4^+^ T cells into earliest activated *Lat*^Y136F^ CD4^+^ T cells (Fig. 5 A). This feature was transmitted to their non-Tfh cell progeny (Fig. 5 A) and was corroborated by the presence of 5.3-fold reduced levels of intracytoplasmic CD3ζ chains in non-Tfh cells from 5-wk-old *Lat*^Y136F^ spleens as compared with their WT counterparts (Fig. 5 B). Considering that CD3ζ limits the expression of TCR–CD3 complexes at the T cell surface (Ardouin et al., 1998; Weissman et al., 1989), it resulted in the expression at the surface of *Lat*^Y136F^ non-Tfh cells of TCR levels that were 17.0-fold reduced as compared with the levels present on WT non-Tfh cells (Fig. 5, B and C). In contrast to *Lat*^Y136F^ non-Tfh cells, *Lat*^Y136F^ Tfh cells retained substantial levels of CD3ζ chain transcripts (Fig. 5 A). As a result, their levels of intracytoplasmic CD3ζ chains were only 2.9-fold reduced as compared with WT Tfh cells, leading to surface TCR levels that were solely 5.2-fold reduced as compared with those present on WT Tfh cells (Fig. 5, B and C). Therefore, commensurate to their reduced expression of CD3ζ chains, non-Tfh and Tfh *Lat*^Y136F^ cells expressed low and intermediate levels of TCR–CD3 complexes at their surface, respectively.

### Functional characterization of *Lat*^Y136F^ Tfh cells

Using in vitro T-B cocultures and adoptive transfer into T cell–deficient hosts, bulk *Lat*^Y136F^ CD4^+^ T cells induced the differentiation of mature B cells into IgG1- and IgE-producing B cells (Genton et al., 2006; Wang et al., 2008). It occurred independently of TCR–MHCII interactions and required CD28 engagement with its CD80/CD86 ligands expressed on B cells (Chevrier et al., 2012; Genton et al., 2006; Wang et al., 2008). To demonstrate that the *Lat*^Y136F^ Tfh cells themselves were responsible for the non-cognate help delivered to B cells by bulk *Lat*^Y136F^ CD4^+^ T cells, they were sorted and co-cultured with CD19^+^ B cells isolated from WT mice and from mice lacking either MHC class II (MHCII^−^) or CD80 and CD86 (CD80^−^CD86^−^) expression. When *Lat*^Y136F^ Tfh cells were cocultured with WT B cells, they induced their differentiation into GL7^+^IgG1^+^ GC B cells (Fig. 5, D and E). Likewise, when *Lat*^Y136F^ Tfh cells were co-cultured with MHCII^−^ B cells, they also induced their differentiation into GL7^+^IgG1^+^ GC B cells with a 1.5-fold reduced potency as compared with WT MHCII^+^ B cells. In contrast, when *Lat*^Y136F^ Tfh cells were cocultured with CD80^−^CD86^−^ B cells, they only triggered the differentiation of minute numbers of GL7^+^IgG1^+^ GC B cells. In control experiment involving WT B cells alone, no GL7^+^IgG1^+^ GC B cells were detected during the culture period. Although *Il4*^high^ non-Tfh cells were the closest to Tfh cells on the basis of transcriptomics, they failed to induce IgG1-producing GC B cells (Fig. 5, D and E). Moreover, congruent with their lack of Tfh cell features, the intriguing latecomer cells also lacked the capacity to induce IgG1-producing GC B cells (Fig. 5, D and E). Therefore, the *Lat*^Y136F^ Tfh cells themselves account for the massive B cell activation, GC center formation, and isotype switch and IgG1 secretion seen in *Lat*^Y136F^ mice. The magnitude of these events is likely accounted for by the presence of 300-fold higher numbers of Tfh cells in 8-wk-old *Lat*^Y136F^ spleens as compared with age-matched WT spleens (Fig. 5 F). Consistent with the above data, a recent analysis of 12-wk-old *Lat*^Y136F^ spleens expressing a reporter detecting IL-4–producing cells showed that *Lat*^Y136F^ Tfh cells were closely located to GC B cells and the sole among *Lat*^Y136F^ CD4^+^ T cells to actively produce IL-4 (O’Brien et al., 2021).

### Characterization of the B cells of *Lat*^Y136F^ spleen

**Figure 6.**
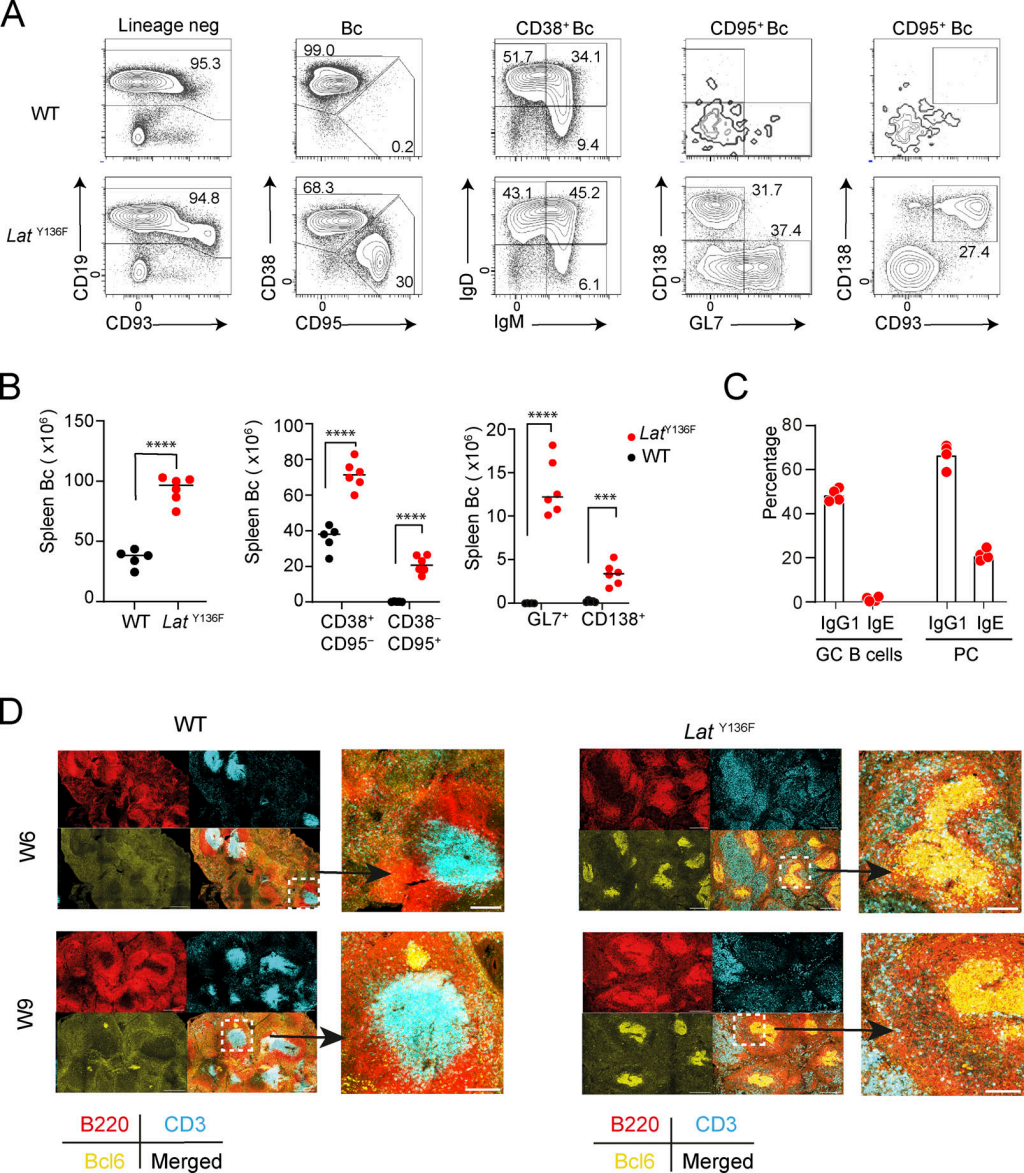
**Characterization of the B cells found in *Lat***^**Y136F**^ **spleen. (A)** CD19^+^ CD93^− to +^ B cells (Bc) from 6–8-wk-old WT and *Lat*^Y136F^ spleen were identified after pregating on live CD45^+^, CD90.2^−^, CD161c^−^, CD11b^−^, CD11c^−^ cells. Mature and activated B cells were further identified using CD38 and CD95, respectively. Among CD95^+^ activated B cells, GL7^+^ GC B cells and CD138^+^ PC can be distinguished. Numbers indicate the percentage of cells in the respective gate. **(B)** Quantification of the data shown in A. Each dot corresponds to a mouse; ***P < 0.002, ****P < 0.001; unpaired Student’s *t* test. Data in A and B are representative of three independent experiments involving each five to six mice. **(C)** B cells from 8-wk-old *Lat*^Y136F^ spleen were subjected to a flow-cytometry procedure that specifically detects intracellular IgE or IgG1, and the percentages of intracellular IgG1^+^ and IgE^+^ GC B cells and PC were determined. Each dot corresponds to a mouse, and the mean is shown. Background staining was established using isotype-matched control antibodies. Data are representative of two experiments involving four mice per condition. **(D)** Spleen sections from 6-wk-old WT and *Lat*^Y136F^ mice were stained with anti-B220-FITC (red), anti-CD3-PE (cyan), and anti-BCL6-AF647 (yellow). Imaging was performed using confocal microscopy at 10× magnification. Scale bars correspond to 300 µm except for the magnification shown at the right, for which scale bars correspond to 100 µm. Data are representative of two experiments with two mice per experiment.

To determine the impact of the *Lat*^Y136F^ Tfh cells on B cells in vivo, we compared the B cell populations found in the spleen of 6–8-wk-old WT and *Lat*^Y136F^ mice (Fig. 6, A and B). *Lat*^Y136F^ spleen contained 2- and 96-fold increased numbers of CD38^+^CD95^−^ follicular B cells and CD38^−^CD95^+^ activated B cells, respectively, as compared with WT spleen. Numerous GL7^+^CD138^−^ GC B cells (13.3 × 10^6^ ±3.2 per spleen) and GL7^−^CD138^+^ PC (3.4 × 10^6^ ±1.2 per spleen) cells were also present in *Lat*^Y136F^ spleens, whereas they were almost absent in WT spleens (Fig. 6 B).

To quantify IgG1- and IgE-expressing B cells in *Lat*^*Y136F*^ spleen, we took into consideration the presence of massive amounts of IgG1 and IgE in *Lat*^Y136F^ serum (Aguado et al., 2002). Accordingly, to avoid detecting IgG1 bound to Fc γ receptor IIb (CD32) or IgE bound to Fc ε receptor II (CD23), two Fc receptors expressed at the B cell surface, we used a flow cytometry procedure that specifically detects the IgE or IgG1 that is located in the intracellular organelles of the secretory pathway (Yang et al., 2012). CD95^+^CD138^−^ GC B cells found in 6–8-wk-old *Lat*^Y136F^ spleen comprised higher percentages of IgG1^+^ cells (48.5%) as compared with IgE^+^ cells (0.38%), whereas 66.6% and 20.8% of the CD95^+^CD138^+^ PC expressed IgG1 and IgE, respectively (Fig. 6 C). Therefore, the *Lat*^Y136F^ Tfh cells had a major impact on B cell activation and differentiation into GC B cells and PC, accounting for the hypergammaglobulinemia G1 and E of *Lat*^Y136F^ mice.

*Lat*^Y136F^ spleens were next imaged by confocal microscopy. Spleen sections from *Lat*^Y136F^ and WT mice were stained with anti-B220 to detect B cells, anti-CD3ε to detect T cells, and anti-BCL6 to detect Bcl6, a transcriptional repressor expressed in GC B cells. 6 wk after birth, most B cell follicles of *Lat*^Y136F^ mice contained GC B cells surrounded by scattered T cells (Fig. 6 D). In contrast, the B cell follicles of WT spleen contained no GC B cells, and the T cells were localized in periarteriolar lymphoid sheaths. In 9-wk-old *Lat*^Y136F^ spleen, every B cell follicle contained GC B cells, and the normal architecture of the spleen was obliterated by large numbers of T cells which likely correspond to latecomer CD4^+^ T cells (Fig. 6 D). 9-wk-old WT spleens showed a distribution of T and B cells similar to that of 6-wk-old WT spleens and contained only a few small GC B cell clusters.

### Characterization of the lymphoid infiltrates of *Lat*^Y136F^ lung

**Figure 7.**
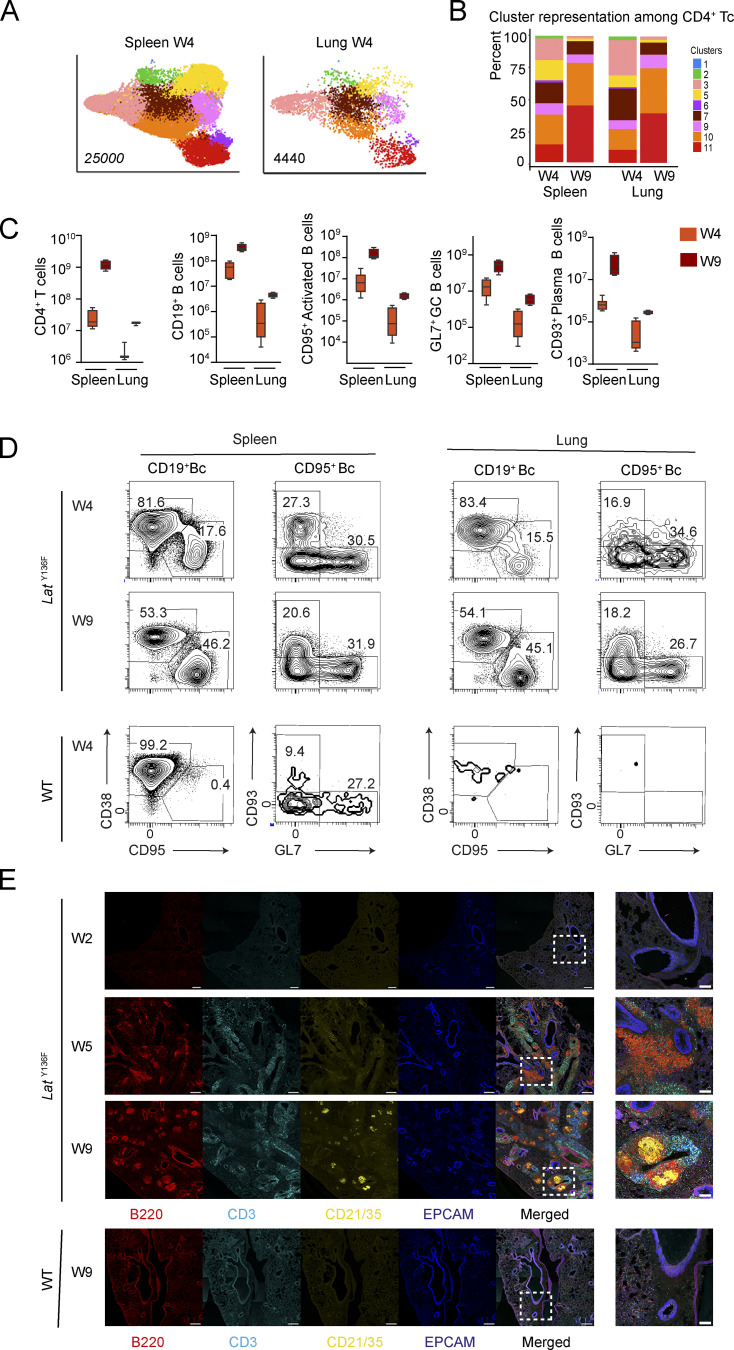
**Characterization of the T and B cells found in *Lat***^**Y136F**^ **lung. (A)** The flow cytometry panel developed on the basis of scRNAseq data was used to compare the CD4^+^ T cells found in the spleen and lung of 4-wk-old *Lat*^Y136F^ mice. **(B)** Contribution (%) of each of the nine identified T cell clusters to the CD4^+^ T cells found in *Lat*^Y136F^ spleen and lung at 4 and 9 wk (W) after birth. Data in B and C are representative of two experiments involving four mice per conditions. **(C)** Numbers of CD4^+^ T cells, CD19^+^ B cells, CD95^+^ activated B cells, GL7^+^ GC B cells, and CD93^+^ PC found in the spleen and lung of 4- and 9-wk-old *Lat*^Y136F^ mice. Three to five mice were analyzed per organ and time point and box plots are shown with the median, boxed interquartile range, and whiskers. **(D)** Flow cytometry analysis of B cells (Bc) from the spleen and lung of *Lat*^Y136F^ and WT mice at 4 and 9 wk after birth. Most of the *Lat*^Y136F^ GL7^−^CD138^+^ PC found in the spleen co-expressed CD93, a C-type lectin expressed during early B cell development and reinduced during PC differentiation (Chevrier et al., 2009; Fig.6 A). In contrast to CD138, CD93 is resistant to the enzymatic cocktail used during lung dissociation procedures. Accordingly, we used it to identify PC among the CD95^+^ activated B cells found in the *Lat*^Y136F^ lung and also in the spleen for the sake of consistency. Data are representative of at least two independent experiments involving two to three mice. **(E)** Lung sections from *Lat*^Y136F^ mice at 2, 5, and 9 wk of age and from WT mice at 9 wk of age were stained with anti-B220-PE (red), anti-CD3 FITC (cyan), anti-CD21/CD35-AF450 (yellow), and anti-EPCAM-AF647 (blue). Imaging was performed using confocal microscopy at 20× magnification. Scale bars correspond to 300 µm except for the magnification shown at the right, for which scale bars correspond to 100 µm. Data are representative of two experiments with two mice per experiment.

Lymphoid infiltrates were previously identified in *Lat*^Y136F^ lung via histopathological examination (Waseda et al., 2021). To determine their cellular composition, we used our flow cytometry panel identifying most of the scRNAseq-defined *Lat*^Y136F^ CD4^+^ T cell clusters (Fig. 4). Prior to preparing lung cell suspensions, the enlarged mediastinal LN were carefully removed and intravenous labeling was performed to gate out vascular T and B cells and focus on those T and B cells residing within the lung parenchyma at the time of analysis (Anderson et al., 2014). Comparative analysis of the lung and spleen of 4-wk-old *Lat*^Y136F^ mice showed that they comprised the very same CD4^+^ T cell clusters and that CD4^+^ CTLs were more represented in the lung, where they corresponded to 25% of the CD4^+^ T cells (Fig. 7, A and B). In contrast, minute numbers of CD8^+^ T cells were detectable in the lung of 4-wk-old *Lat*^Y136F^ mice. As already documented in *Lat*^Y136F^ spleen, the increased numbers of CD4^+^ T cells found in 9-wk-old *Lat*^Y136F^ lung were dominated by latecomer CD4^+^ T cells (Fig. 7, B and C). Up to 0.3 × 10^6^ CD4^+^ CTL and 0.4 × 10^6^ Tfh cells were, however, still present per 9-wk-old *Lat*^Y136F^ lung. Comparative analysis of the B cells found in the spleen and lung of 4- and 9-wk-old *Lat*^Y136F^ mice showed that CD38^−^CD95^+^ activated B cells, GL7^+^CD138^−^ GC B cells, and GL7^−^CD138^+^ PC steadily increased during the observation period (Fig. 7, C and D). 9 wk after birth, the *Lat*^Y136F^ spleen and lung contained 21.8 × 10^6^ ±6.2 and 0.2 × 10^6^ ±0.05 PC, respectively (Fig. 7 C).

Lung sections from *Lat*^Y136F^ mice at 2, 5, and 9 wk of age were stained with anti-B220, anti-CD3ε, and anti-EPCAM to detect lung epithelial cells, and anti-CD21/CD35 to detect follicular dendritic cells (FDC), and then imaged by confocal microscopy. 2 wk after birth, no T and B cells were observed (Fig. 7 E). In contrast, 5 wk after birth, large peribronchial and perivascular T and B cell infiltrates were observed, containing B cell clusters that were not associated with FDC network. Consistent with flow-cytometry analysis (Fig. 7, C and D), larger T and B cell infiltrates were present 9 wk after birth, and FDC networks were found in most B cell clusters (Fig. 7 E). Such tertiary lymphoid structures are reminiscent of those found in the lymphoid infiltrates of the lacrimal and submandibular glands of IgG4-RD patients (Ebbo et al., 2012). Therefore, when analyzed at the single-cell level, the lymphoplasmacytic infiltrates found in the lung of *Lat*^Y136F^ mice resembled those found in the affected tissues of IgG4-RD patients.

### Role of B cells in the unfolding of *Lat*^Y136F^ DLSP

**Figure 8.**
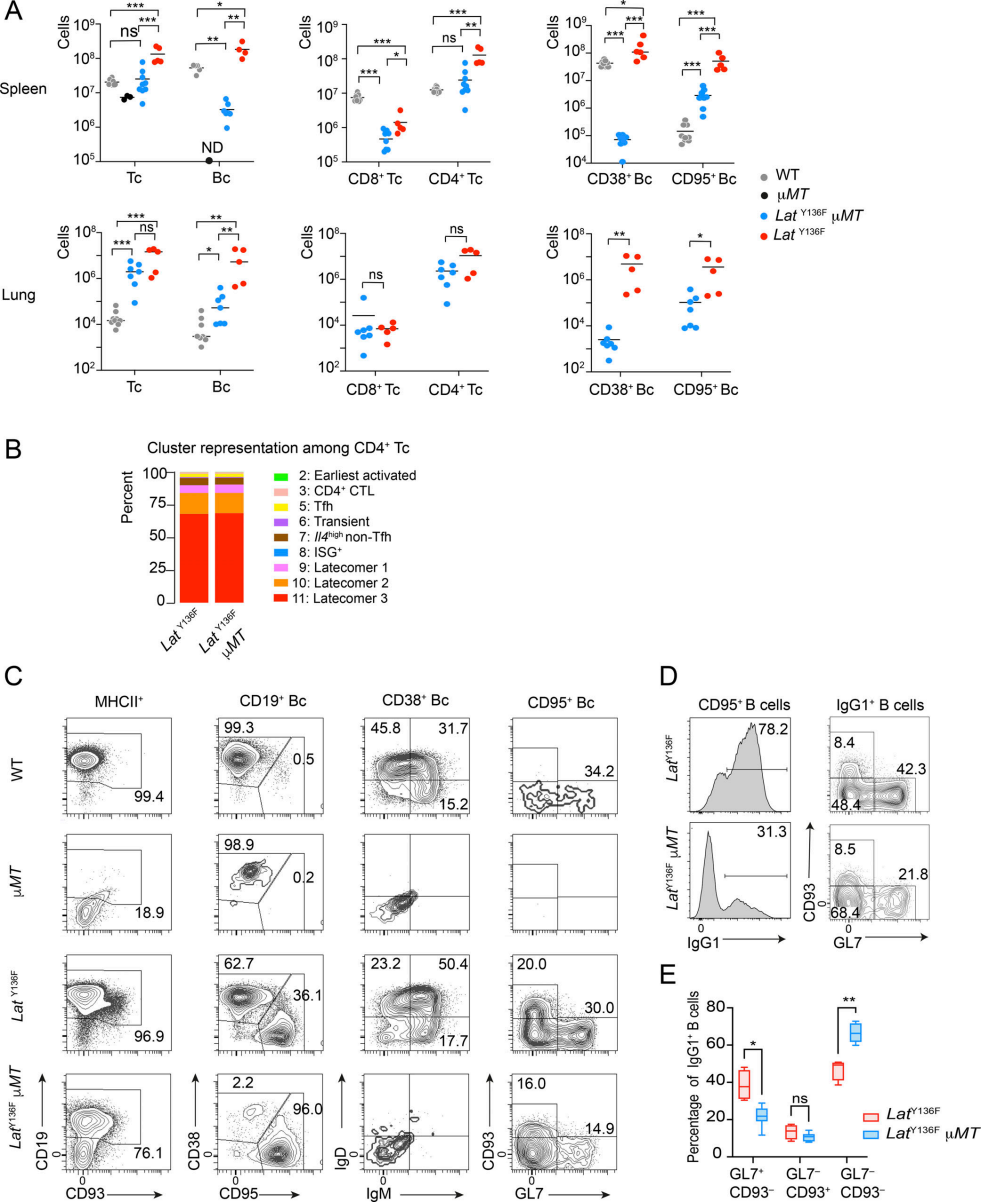
**T cell development in *Lat***^**Y136F**^ **mice lacking B cells. (A)** Absolute number of T (Tc) and B cells (Bc; left), CD4^+^ and CD8^+^ T cells (middle), and CD38^+^ mature and CD95^+^ activated B cells (right) in the spleen (top) and lung (bottom) of 5–6-wk-old WT, *Lat*^Y136F^, μMT, and *Lat*^Y136F^ μMT mice. Data in A–C are representative of three independent experiments and correspond to five to nine individual mice per genotype except in WT mice, for which three mice were analyzed. Each dot corresponds to a mouse, and the mean is shown. *P 0.01, **P 0.002, ***P 0.0001, ns, non-significant; unpaired Student’s *t* test. **(B)** Contribution (%) of each of the nine identified T cell clusters (see key) to the CD4^+^ T cells found in 5–6-wk-old *Lat*^Y136F^ and *Lat*^Y136F^ μMT spleen. Six mice were analyzed per genotype, and the mean is shown. **(C)** Flow cytometry analysis of splenic B cells in 5–6-wk-old WT, μMT, *Lat*^Y136F^, and *Lat*^Y136F^ μMT mice. A gating strategy identical to the one shown in Fig. 6 A was used. Numbers indicate the percentage of cells in the specified windows. **(D)** CD95^+^ B cells from 8-wk-old *Lat*^Y136F^ and *Lat*^Y136F^ μMT mice spleen were analyzed by flow cytometry for the expression of IgG1. The percentage of IgG1^+^ CD95^+^ B cells is shown. **(E)** IgG1^+^ CD95^+^ B cells were analyzed for the expression of CD93 and GL7. The percentage of cells found within each of the specified gates is indicated. *P < 0.02, **P < 0.002; unpaired Student’s *t* test. In D and E, data are representative of two experiments involving each five to seven mice.

Disruption of one of the membrane exons of the μ heavy chain constant region gene in μMT mice prevented IgM surface expression and in turn the development of mature B cells (Kitamura et al., 1991). Therefore, *Lat*^Y136F^ mice were crossed with μMT mice to assess the contribution of B cells to the *Lat*^Y136F^ DLSP. The spleen and lung of 5–6-wk-old *Lat*^Y136F^ μMT mice were analyzed by flow cytometry and compared with age-matched *Lat*^Y136F^ and μMT littermates (Fig. 8 A). As expected, the spleen of μMT mice contained no detectable mature B cells whereas *Lat*^Y136F^ spleen contained increased numbers of CD38^+^ and CD95^+^ B cells as compared with WT spleen. Some B cells were unexpectedly found in the spleen of *Lat*^Y136F^ μMT mice, in numbers, however, 56-fold reduced as compared with *Lat*^Y136F^ spleens, respectively (see below). The markedly reduced B cell numbers found in *Lat*^Y136F^ μMT spleen were associated with 5.3- and 3-fold decreased numbers of CD4^+^ and CD8^+^ T cells, respectively, as compared with *Lat*^Y136F^ spleen, and a similar trend was observed in the lung for CD4^+^ T cells (Fig. 8 A). The reduced numbers of B cells found in *Lat*^Y136F^ μMT mice had, however, no impact on *Lat*^Y136F^ CD4^+^ T cell diversification (Fig. 8 B).

Overexpression of Bcl-2 or inactivation of the Fas-mediated apoptosis pathway in μMT mice prolonged the life span of pro-B cells allowing their inefficient T cell–dependent differentiation into mature B cells expressing membrane-bound IgH chain isotypes other than IgM (Hasan et al., 2002; Lutz et al., 1998; Melamed et al., 2000; Orinska et al., 2002; Tarlinton et al., 1997). Likewise, the spleen of 5–6-wk-old *Lat*^Y136F^ μMT mice contained mature CD19^+^ B cells that lacked IgD and IgM at their surface (Fig. 8 C). They had a CD38^−^CD95^+^ activated B cell phenotype and a quarter of them corresponded to GL7^+^CD93^−^ GC B cells and GL7^−^CD93^+^ PC. 31.3% of those CD95^+^ activated B cells expressed IgG1, suggesting that switching to downstream IgH isotypes such as IgG1 contributed to the partial rescue of B cell development observed in the *Lat*^Y136F^ μMT mice (Fig. 8, D and E). Therefore, *Lat*^Y136F^ Tfh cells were unexpectedly capable of inducing the differentiation of μMT pro-B cells into CD19^+^ activated B cells corresponding to GC B cells and PC.

### Triggers and modifiers of the *Lat*^Y136F^ DLSP

**Figure 9.**
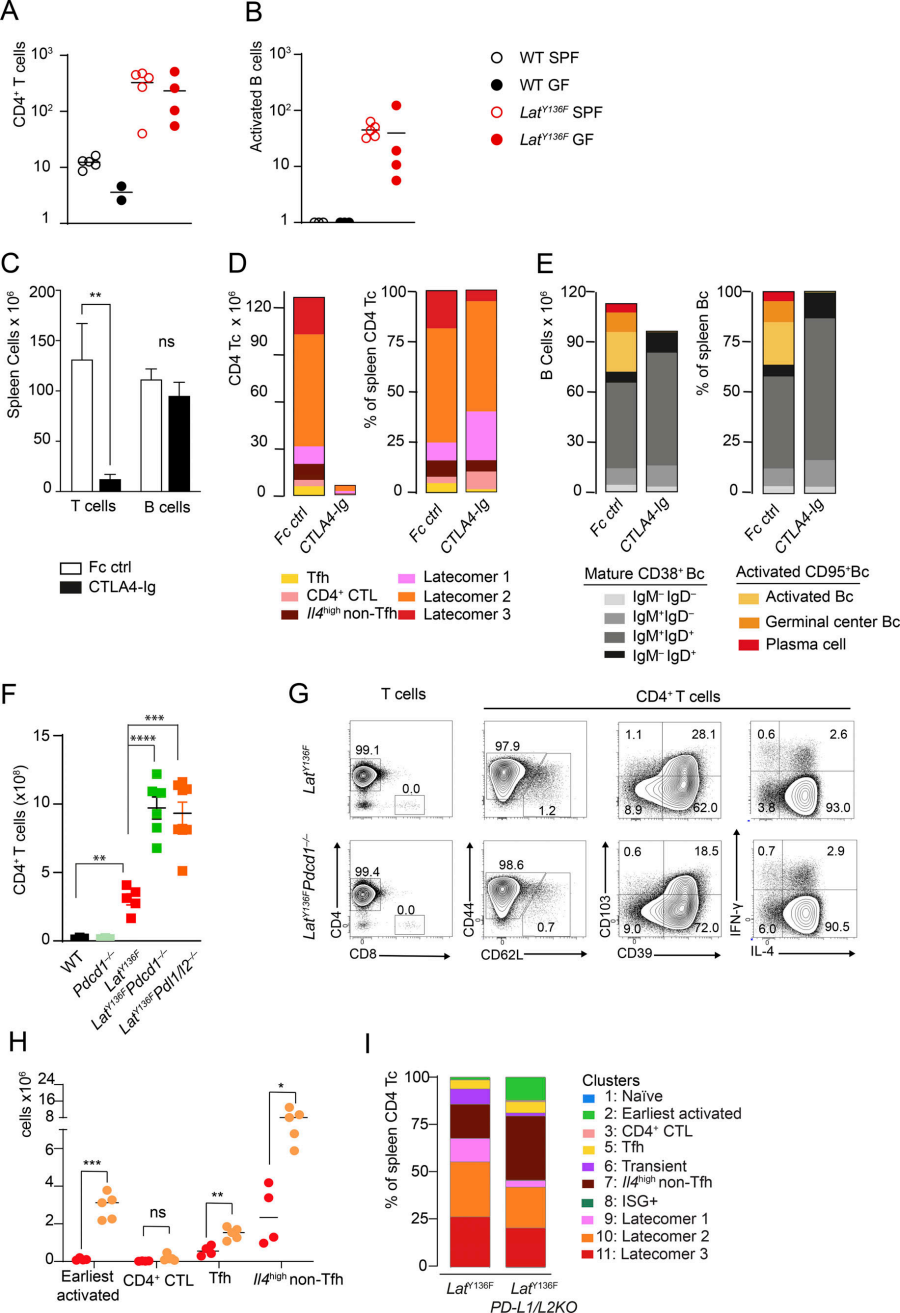
**The *Lat***^**Y136F**^ **DLSP unfolds under germ-free condition, is blocked by CTLA-4-Ig, and is exacerbated in absence of PD-1 engagement. (A)** Numbers of CD4^+^ T cells found in the spleen of 6–8-wk-old WT and *Lat*^Y136F^ mice raised under specific pathogen–free (SPF) or germ-free (GF) conditions. Each dot corresponds to a mouse, and the mean is shown. Data in A and B are representative of two experiments involving at least two mice per condition. **(B)** Numbers of CD95^+^ activated B cells found in the spleen of 6–8-wk-old WT and *Lat*^Y136F^ mice raised under SPF or GF conditions. Each dot corresponds to a mouse, and the mean is shown. **(C)** Numbers of T (Tc) and B cells (Bc) in the spleen of 5-wk-old *Lat*^Y136F^ mice that have received either CTLA-4-Ig or IgG1 Fc control. T cells: **P < 0.003, B cells: P < 0.57; two-way ANOVA test. Data in C–E are representative of two independent experiments involving three mice per condition, and the mean and SD are shown. **(D)** Contribution in absolute numbers and percentages of each of the six specified T cell clusters (see key) to the CD4^+^ T cells found in the spleen of 5-wk-old *Lat*^Y136F^ mice that have received either CTLA-4-Ig or IgG1 Fc control. **(E)** Contribution in absolute numbers and percentages of each of the specified mature and activated B cell subsets (see key) to the B cells of the spleen of 5-wk-old *Lat*^Y136F^ mice that have received either CTLA-4-Ig or IgG1 Fc control. **(F)** Numbers of CD4^+^ T cells in the spleen of 8-wk-old WT (three individuals), *Lat*^Y136F^ (five individuals), *Pdcd1*^**−/−**^ (three individuals), *Lat*^Y136F^ *Pdcd1*^**−/−**^ (six individuals), and *Lat*^Y136F^ *Pdl1/2*^**−/−**^ (eight individuals) mice. Each dot corresponds to a mouse, and the mean and SD are indicated. Data in F are representative of three independent experiments. WT versus *Lat*^Y136F^: **P < 0.002, *Lat*^Y136F^vs. *Lat*^Y136F^ *Pdcd1*^**−/−**^: ****P < 0.0001, *Lat*^Y136F^ versus *Lat*^Y136F^ *Pdl1/2*^**−/−**^: ***P < 0.001; multiple unpaired Student’s *t* test. **(G)** Spleens of 6–8-wk-old *Lat*^Y136F^ and *Lat*^Y136F^ *Pdcd1*^**−/−**^ mice were analyzed by flow cytometry for the specified pairs of markers. Also shown is IL-4 and IFN-γ production after stimulation with PMA and ionomycin for 4 h in the presence of monensin. **(H)** Comparison of the numbers of earliest activated CD4^+^ cells, CD4^+^ CTL, Tfh cells, and *Il4*^high^ non-Tfh cells found in the spleen of *Lat*^Y136F^ and *Lat*^Y136F^ *Pdl1/2*^**−/−**^ mice at 4 wk after birth. Earliest activated CD4^+^ cells: ***P < 0.01, CD4^+^ CTL: ns, Tfh cells: **P < 0.002, *Il4*^high^ non-Tfh cells: *P < 0.005; multiple unpaired Student’s *t* test. **(I)** Contribution (%) of each of the 10 identified CD4^+^ T cell clusters to the CD4^+^ T cell in *Lat*^Y136F^ and *Lat*^Y136F^ *Pdl1/2*^**−/−**^ spleen at 4 wk after birth (see key). Data in H and I are representative of three independent experiments involving a total of four to five individual mice per genotype.

MHCII-restricted self-peptides are thought to trigger the autoreactive TCR expressed by the naive *Lat*^Y136F^ CD4^+^ T cells that seed the SLO (Aguado et al., 2002; Sommers et al., 2005). To determine whether peptides derived from commensal microbiota contribute to such activation, we compared *Lat*^Y136F^ mice raised under specific pathogen–free or germ-free conditions and found that the *Lat*^Y136F^ DLSP developed irrespective of the presence of commensal microbiota (Fig. 9, A and B). Therefore, the *Lat*^Y136F^ DLSP can unfold under germ-free conditions, supporting the view that the autoreactive TCR expressed by naive *Lat*^Y136F^ CD4^+^ T cells primarily react against self-peptides.

CD28 is ubiquitously expressed by *Lat*^Y136F^ CD4^+^ T cells (Data S3) and is essential for their expansion (Mingueneau et al., 2009). To solidify this view, 2-wk-old *Lat*^Y136F^ mice were weekly injected for three consecutive weeks with CTLA-4-Ig fusion protein that prevents CD28 engagement by blocking CD80 and CD86 availability. The massive *Lat*^Y136F^ CD4^+^ T cell expansion occurring between 2 and 5 wk after birth was 10.5-fold reduced by CTLA-4-Ig treatment (Fig. 9 C). Analysis of the composition of the remaining CD4^+^ T cells showed that the differentiation of Tfh cells was proportionally more affected by CTLA-4-Ig treatment (Fig. 9 D), explaining the lack of activated B cells in the spleen of CTLA-4-Ig–treated *Lat*^Y136F^ mice (Fig. 9 E).

*Lat*^Y136F^ spleen contains PD-1^high^ CXCR5^+^ Tfh cells (cluster 5) and PD-1^int to low^ CXCR5^−^ T cells (clusters 2, 3, and 7; Fig. 5 F), prompting us to determine whether the *Lat*^Y136F^ DLSP was subjected to PD-1 coinhibition. *Lat*^Y136F^ mice lacking either PD-1 (*Lat*^Y136F^ *Pdcd1*^**−/−**^ mice) or the two PD-1 ligands, known as PD-L1 and PD-L2 (encoded by the *Cd274* and *Pdcd1lg2* gene, respectively; *Lat*^Y136F^ *Pdl1/2*^**−/−**^ mice), were developed. The spleens of 8-wk-old *Lat*^Y136F^ *Pdcd1*^**−/−**^ and *Lat*^Y136F^ *Pdl1/2*^**−/−**^ mice contained close to 1 billion CD4^+^ T cells, corresponding to a 3.2- and 53-fold increased cellularity as compared with age-matched *Lat*^Y136F^ and WT spleens, respectively (Fig. 9 F). Analysis of the spleen of *Lat*^Y136F^ and *Lat*^Y136F^ *Pdl1/2*^**−/−**^ mice at 4 wk after birth and prior to the massive latecomer T cell expansion permitted readily visualizing the nine T cell clusters defined by flow cytometry.  It showed that the lack of PD-1 engagement had no impact on their type 2 polarization (Fig. 9 G). It affected, however, cluster representation in that the percentage of earliest activated cells (cluster 2) was increased 27-fold as compared with *Lat*^Y136F^ mice, whereas those of Tfh cells (cluster 5) and *Il4*^high^ non-Tfh cells (cluster 7) increased 2.6- and 3.4-fold, respectively (Fig. 9, H and I). Therefore, PD-1 negatively controls the expansion of the PD-1^int to high^ *Lat*^Y136F^ CD4^+^ T cell clusters and its absence exacerbated the magnitude of the *Lat*^Y136F^ DLSP.

## Discussion

**Figure 10.**
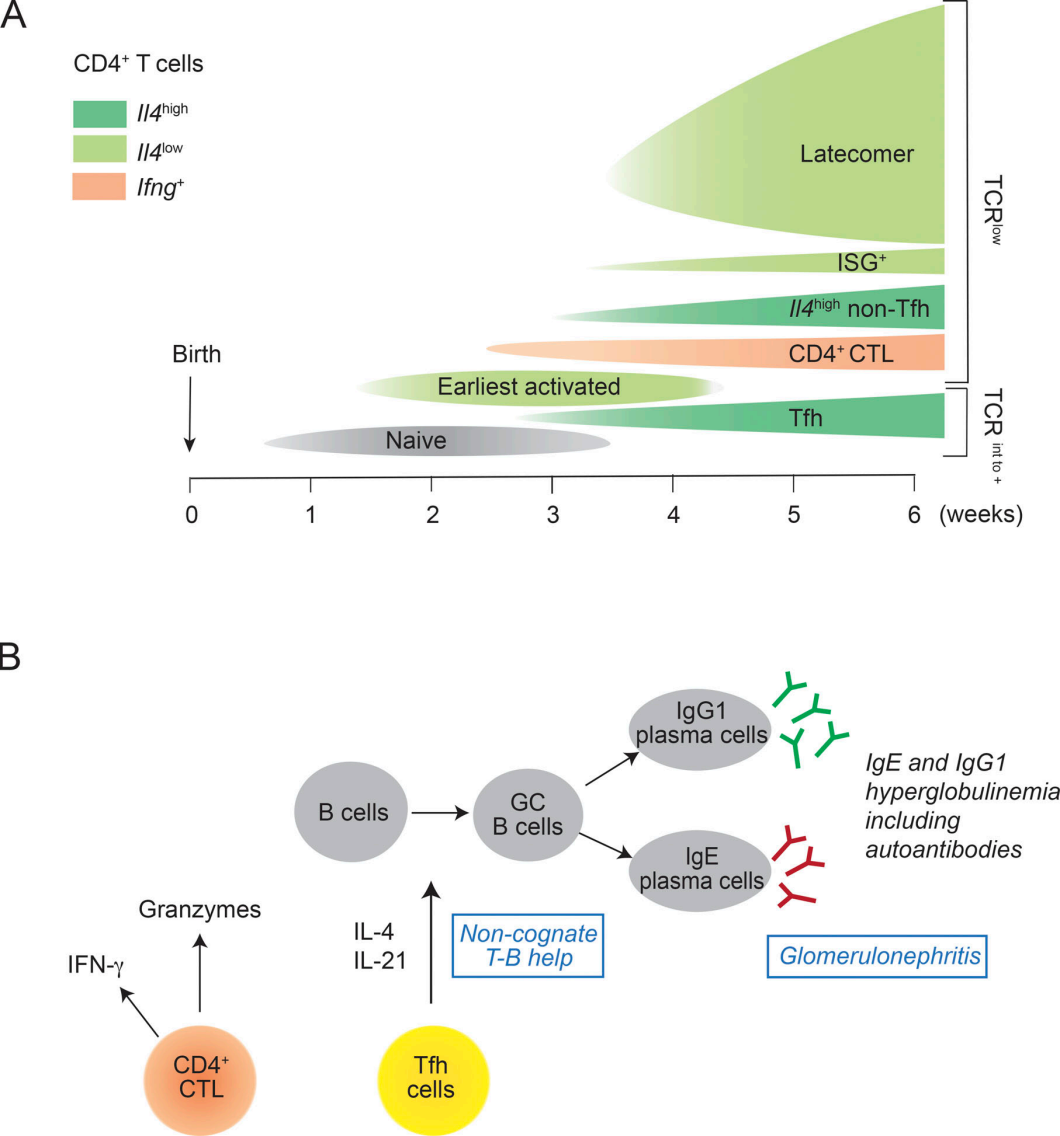
**Model summarizing the timeline of *Lat***^**Y136F**^ **CD4**^**+**^ **T cell diversification and the composition of the *Lat***^**Y136F**^ **lung infiltrates. (A)** Timeline of CD4^+^ T cell diversification in *Lat*^Y136F^ spleen. The expression of *Il4* and *Ifng* transcripts (see key) and the surface TCR levels (see right side) are specified. **(B)** Cellular composition of the *Lat*^Y136F^ lung infiltrates emphasizing their similarity with the lymhoplasmacytic infiltrates observed in the affected tissues of IgG4-RD patients prior to the expansion of latecomer *Lat*^Y136F^ CD4^+^ T cells. As demonstrated here, the *Lat*^Y136F^ DLSP primarily results from a defect intrinsic to conventional CD4^+^ T cells that is likely precipitated by the lymphopenic environment in which it starts and by the absence of functional Treg cells (Chuck et al., 2010; Martin et al., 2017; Wang et al., 2008; Yi et al., 2019).

Using scRNAseq and flow cytometry, we provided here the most comprehensive analysis yet on the composition of the T and B cell populations found in the spleen and lung of *Lat*^Y136F^ mice and responsible for the DLSP (Fig. 10). We also unveiled the molecular and cellular events that trigger the onset of the *Lat*^Y136F^ DLSP and showed that it can unfold in absence of microbial inputs. During intrathymic T cell development, expression of partially functional *Lat*^Y136F^ signalosomes allows the selection of small numbers of overtly self-reactive CD4^+^ T cells that egress to the periphery (Aguado et al., 2002; Sommers et al., 2005). They correspond to cluster 1 cells and express transcriptome and surface TCR levels similar to naive WT CD4^+^ T cells. Following engagement of their self-reactive TCR, the signals delivered by their intact TCR triggering module made of the TCR-CD3 complex and of the LCK and ZAP-70 protein tyrosine kinases are inappropriately decoded by the malfunctioning LAT signalosomes, resulting in their conversion into earliest activated CD4^+^ T cells (cluster 2) that express decreased CD3ζ chain transcripts and proteins. Consistent with our scRNAseq analysis of the *Lat*^Y136F^ CD4^+^ DLSP onset, flow cytometry analysis of the SLO of 2-wk-old *Lat*^Y136F^ mice expressing a green fluorescent protein (GFP) reporter identifying cells with actively transcribed *Il4* genes showed that they contained both GFP^−^*Il4*^−^ CD4^+^ T cells expressing physiological TCR levels and converted GFP^+^*Il4*^+^ CD4^+^ T cells with greatly diminished surface TCR levels (Mingueneau et al., 2009). This seminal “conversion” event at the basis of the *Lat*^Y136F^ DLSP suggests that in activated WT T cells, proper transcription of the gene coding for CD3ζ chains is not constitutive and requires continuous reinforcement by a functional TCR–LAT signaling axis, as previously suggested for naive T cell homeostasis (Markegard et al., 2011; Myers et al., 2017). Interestingly, the TCR unresponsiveness characteristics of some chronic autoimmune diseases such as systemic lupus erythematosus have been also associated with the presence of reduced levels of CD3ζ chain (Liossis et al., 1998; Nambiar et al., 2001; Takeuchi et al., 2012; Zhang et al., 2007).

Naive WT CD4^+^ T cells engineered in vitro to express LAT^Y136F^ molecules in lieu of WT LAT molecules keep normal surface levels of TCR–CD3 complexes for a few days. It permitted demonstrating that when expressed at physiological level, their TCR triggering module is fully functional and that it is the LAT^Y136F ^molecules themselves that prevent TCR-mediated Ca^2+^ influx, NFAT activation, and PLCγ1-driven activation of the ERK pathway (Mingueneau et al., 2009; Shen et al., 2010). Such unresponsiveness to TCR signals should be even more exacerbated in the case of *Lat*^Y136F^ Tfh cells, which additionally express 5.2-fold reduced levels of TCR as compared with WT Tfh cells. Therefore, the reliance of *Lat*^Y136F^ Tfh cells on the delivery of non-cognate, CD28-dependent help to B cells permits to obviate their co-expression of reduced surface levels of autoreactive TCR and of malfunctioning LAT signalosomes. Due to this unique feature, *Lat*^Y136F^ Tfh cells induced a polyclonal B cell activation resulting in massive amounts of IgG1 and IgE antibodies among which the proportionate presence of autoantibodies was responsible for systemic autoimmunity (Genton et al., 2006). Akin to the atypical *Lat*^Y136F^ Tfh cells, those found in a few other mouse models were also capable of providing help to B cells independent of TCR engagement (Biram and Shulman, 2020; Nijnik et al., 2006). When considered together with the 17.0-fold reduced levels of TCR–CD3 complexes expressed at the surface of *Lat*^Y136F^ non-Tfh cells, our results explain that the pathological manifestations characteristic of *Lat*^Y136F^ DLSP and mediated by both Tfh and non-Tfh cells unfold irrespective of TCR–MHCII interactions (Chevrier et al., 2012; Mingueneau et al., 2009; Wang et al., 2008).

In *Lat*^Y136F^ μMT mice, the presence of Tfh cells capable of helping B cells in a non-cognate mode likely accounted for the development of a few B cells that had switched to the IgG1 isotype to bypass the lack of IgM. Their unexpected presence makes it difficult to conclude whether B cells are required for the development of the *Lat*^Y136F^ DLSP. The B cell numbers found in *Lat*^Y136F^ μMT spleens were, however, 56-fold reduced as compared with age-matched *Lat*^Y136F^ spleens, and it resulted in a 5.3-fold decrease in the numbers of CD4^+^ T cells in *Lat*^Y136F^ μMT spleen as compared with age-matched *Lat*^Y136F^ spleens. When considered together with a recent report (O’Brien et al., 2021), it suggests that the CD28 ligands expressed at the surface of B cells contribute to drive the activation and proliferation of pathogenic *Lat*^Y136F^ CD4^+^ T cells. Such findings in *Lat*^Y136F^ μMT mice are reminiscent of the marked reduction of Tfh cells and CD4^+^ CTL numbers observed in IgG4-RD patients following B cell depletion with the anti-CD20 antibody rituximab (Katz and Stone, 2022; Perugino and Stone, 2020). Altogether, those observations suggest that B cells play a role in the etiopathogenesis of IgG4-RD and *Lat*^Y136F^ DLSP by displaying antigens and CD28 ligands, respectively.

As demonstrated for the *Lat*^Y136F^ mutation, several partial-loss-of-function mutations impede TCR signaling and paradoxically trigger the development of effector T cells responsible for autoimmunity and chronic inflammation (Altin et al., 2011; Liston et al., 2008). For instance, partial-loss-of-function mutations in ZAP-70 reduce T cell development and lead to the selection of a smaller TCR pool with greater self-reactivity (Ashouri et al., 2021; Tanaka et al., 2023). Those ZAP-70 mutations are proportionately more detrimental for Treg cell development and function than for those of conventional CD4^+^ effector T cells, leading to the expansion of pathogenic autoreactive CD4^+^ T cells. Importantly, in the ZAP-70–based models of autoimmune inflammation, both the generation and function of the autoreactive CD4^+^ T cells critically depend on TCR signaling. In marked contrast, following the first engagement of their TCR with self-antigens, the T cell signal-transduction network of *Lat*^Y136F^ CD4^+^ T cells adopts a novel wiring over a few days, the architecture of which remains to be elucidated, in which the TCR hands over its central role in T cell activation to CD28. Therefore, after such an initial TCR-dependent step, all the subsequent manifestations of the *Lat*^Y136F^ DLSP, including the production of autoantibodies, relied on CD28 engagement (Chevrier et al., 2012; Mingueneau et al., 2009; Wang et al., 2008), a view reinforced here by our demonstration that those manifestations can be blocked via early CTLA-4-Ig treatment. When combined with the absence of functional *Lat*^Y136F^ Treg cells (Chuck et al., 2010; Wang et al., 2008), the non-cognate, “innate-like” activation properties of *Lat*^Y136F^ CD4^+^ T cells explain the fast unfolding of the *Lat*^Y136F^ DLSP. Interestingly, the CD4^+^ Th2 cells found in a condition related to IgG4-RD and called “lymphocytic variant of human hypereosinophilic syndrome” are reminiscent of activated *Lat*^Y136F^ CD4^+^ T cells in that they express low levels of TCR at their surface and primarily respond to CD28 signals (Carpentier et al., 2020; Roufosse et al., 1999). Therefore, despite falling within the same signaling pathway, mutations affecting either the TCR triggering module (as illustrated by ZAP-70) or the LAT-based TCR signal diversification module cause immune dysregulation via markedly distinct molecular and cellular mechanisms.

The *Lat*^Y136F^ CD4^+^ T cells were parsed out using single-cell transcriptomics into 11 CD4^+^ T cell clusters (Fig. 10 A). Although the *Lat*^Y136F^ CD4^+^ T cell diversification occurred independently of physiological TCR signals, three of the identified effector T cell clusters (Tfh, CD4^+^ CTL, and ISG^+^ CD4^+^ T cells) can be reliably assigned to canonical CD4^+^ effector T cell types (Fig. 10). CD4^+^ CTLs have been previously identified in human with chronic viral infections (Appay et al., 2002), hepatocellular carcinoma (Zheng et al., 2017), and autoimmune fibrotic diseases, including IgG4-RD (Allard-Chamard et al., 2021; Maehara et al., 2017; Mattoo et al., 2016, 2017; Yabe et al., 2021). CD4^+^ CTLs are also expanded in healthy supercentenarians and contribute to clearing senescent cells and delaying the aging of the skin (Hasegawa et al., 2023). ISG^+^ CD4^+^ T cells have been previously found in the blood and kidneys of patients with lupus nephritis (Arazi et al., 2019; Szabo et al., 2019; Wang et al., 2022), and in mouse during chronic viral infections (Crawford et al., 2014), allergic airway inflammation (Tibbitt et al., 2019), and colonic infection (Kiner et al., 2021). A recent single-cell transcriptomics–based study of the peripheral blood mononuclear cells found in IgG4-RD patients showed that a subset of CD4^+^ central memory T cells also expressed ISG (Wu et al., 2022). The role of ISG^+^ CD4^+^ T cells in the etiology of IgG4-RD and *Lat*^Y136F^ DLSP remains, however, to be established. Therefore, by providing an unprecedented view of the composition of the splenic and lung T cell populations associated with the onset and establishment of the *Lat*^Y136F^ DLSP, our analysis demonstrated that all the cell types (Tfh cells, CD4^+^ CTL, and IgG1-producing B cells) causative of IgG4-RD disease are present in *Lat*^Y136F^ SLO and lung lymphoplasmacytic infiltrates.

Clusters 9, 10, and 11 were coined “latecomer” CD4^+^ T cells based on their kinetics of appearance. As recently illustrated for some gut CD4^+^ T cell clusters (Kiner et al., 2021), they do not readily conform to previous CD4^+^ T cell effector archetypes. They express the highest levels of colony-stimulating factor 1 transcripts among the 11 clusters, and upon expansion, they obliterated the normal architecture of SLO. Latecomer cells expressed the cysteinyl leukotriene receptor 1 (CYSLTR1), and due to their overrepresentation in 6-wk-old spleen, our former bulk RNAseq analysis of *Lat*^Y136F^ CD4^+^ T cells mistakenly suggested that *Cystlr1* expression constituted a generic feature of the *Lat*^Y136F^ CD4^+^ T cells (Prinz et al., 2005). Importantly, the manifestations of *Lat*^Y136F^ DLSP started 2 wk after birth (Archambaud et al., 2009; Mingueneau et al., 2009; and this study), irrespective of the presence of latecomer CD4^+^ T cells, and their contribution to the *Lat*^Y136F^ DLSP remains thus to be established.

When looking at clinical manifestations and histopathological findings, the Lat^Y136F^ DLSP exhibits many features similar to IgG4-RD (see Introduction and Fig. 10 B). Moreover, when considering causative cellular disease drivers, both the *Lat*^Y136F^ DLSP and IgG4-RD are characterized by the accumulation in the SLO and lung of both Tfh cells capable of inducing IgG1/IgG4, and IgE autoantibodies causing systemic autoimmunity and of CD4^+^ CTL that by triggering apoptosis contributes to promote fibrosis and organ dysfunction (Chen et al., 2019; Katz and Stone, 2022; Maehara et al., 2023; Munemura et al., 2022; Perugino and Stone, 2020). Akin to the results reported here for the *Lat*^Y136F^ DLSP, treatment of IgG4-RD with CTLA-4-Ig (Abatacept) also reduced IgG4 and IgE levels (Matza et al., 2022), emphasizing the contribution of CD28 co-stimulation to both *Lat*^Y136F^ DLSP and IgG4-RD. In contrast to IgG4-RD where the average age at diagnosis is in the fifties (Katz and Stone, 2022), the mouse *Lat*^Y136F^ DLSP develops over 6–8 wk after birth. Oligoclonal CD4^+^ expansions have been observed in the blood of IgG4-RD patients and postulated to result from the expression of TCR specific for self-antigens (Mattoo et al., 2016, 2017). In contrast, the converted *Lat*^Y136F^ CD4^+^ T cells are primarily triggered by CD28 and they help B cells in a quasi-mitogenic manner. Therefore, although the histopathological manifestations and constellation of pathogenic lymphoid cells at play in *Lat*^Y136F^ DLSP and IgG4-RD are strikingly similar, the underlying molecular malfunctions intrinsic to conventional CD4^+^ T cells and responsible for the etiology of the *Lat*^Y136F^ DLSP and IgG4-RD likely differ.

Although the *Lat*^Y136F^ Tfh cells had a strong type 2 bias, the presence of high levels of IgE is not a universal feature of active IgG4-RD (Perugino et al., 2021). Moreover, no increase in IgG4 or IgE is observed in four other inflammatory fibrotic conditions that display T and B cell infiltrates similar to those seen in IgG4-RD and *Lat*^Y136F^ DLSP and correspond to fibrosing mediastinitis (Allard-Chamard et al., 2021), systemic sclerosis (Maehara et al., 2020), Grave’s orbitopathy (Zhang et al., 2023), and severe COVID-19 (Kaneko et al., 2022; Perugino et al., 2021). Therefore, the pathological manifestations observed in *Lat*^Y136F^ mice might have relevance for inflammatory fibrotic diseases beyond IgG4-RD. Along that line, we previously showed that STAT6 deletion converts the *Lat*^Y136F^ DLSP into a type 1 inflammatory and autoimmune disorder involving Th1 and CD8^+^ effector T cells (Archambaud et al., 2009). Further analysis via single-cell transcriptomics of the T and B cell found in the SLO and the lung infiltrates of *Lat*^Y136F^ mice deficient in STAT6 will permit determining whether they constitute better mouse mimics of the human inflammatory fibrotic conditions that are not associated with the presence of IgG4 or IgE.

In conclusion, regardless of the notable differences existing between IgG4-RD and *Lat*^Y136F^ DLSP, the early onset, complete penetrance, and magnified pathological manifestations of the *Lat*^Y136F^ DLSP should facilitate therapeutic target discovery and the preclinical evaluation of drug candidates intending to treat IgG4-RD, type 2 immune disorders, and inflammatory fibrotic conditions. Along that line, the *Lat*^Y136F^ mouse model already permitted to demonstrate that activated Th2 cells functionally express CYSLTR1 (Prinz et al., 2005) to identify CARMIL2/RLTPR as a cytosolic protein essential for CD28 costimulation (Liang et al., 2013; Roncagalli et al., 2016; Wang et al., 2016) and to evaluate therapeutics such as Irbesartan (Cui et al., 2019), CTLA4-Ig (this paper), and anti-thymic stromal lymphopoietin antibody (Lu et al., 2022). Although the link between partial immunodeficiency and autoimmune inflammation has long been appreciated in human and mouse, most of the underlying molecular mechanisms remain elusive. By providing a more complete model of the *Lat*^Y136F^ DLSP etiology, our results suggest that mutations affecting the LAT-based TCR signal diversification module or the ZAP-70–based TCR triggering module cause T cell–mediated autoimmune inflammation via markedly distinct molecular and cellular mechanisms.

## Materials and methods

### Mice

Mice were on a C57BL/6 background and maintained in specific pathogen–free conditions. *Lat*^*Y136F*^ (Liang et al., 2013), Cd80^−/−^Cd86^−/−^ (Borriello et al., 1997), MHCII-deficient (Madsen et al., 1999), *Cd274*^−/−^/*Pdcd1lg2*^−/−^ (Keir et al., 2007), and *Ighm*^tm1Cgn^ (Kitamura et al., 1991) mice have been described. *Ighm*^tm1Cgn^ mice are also known as μMT mice. *Pdcd1*^−/−^ (http://www.informatics.jax.org/reference/allele/MGI:6435725) were kindly provided by Rene De Waal Malefyt (Merck Research Laboratory, Palo Alto, CA, USA). Germ-free *Lat*^Y136F^ mice were obtained from TAAM-PHENOMIN, transported under sterile conditions, and immediately analyzed on arrival.

### Animal experimental guidelines

Mice were handled in accordance with national and European laws for laboratory animal welfare and experimentation (EEC Council Directive 2010/63/EU, September 2010), and protocols were approved by the Marseille Ethical Committee for Animal Experimentation and French Animal Ethics Committee (approval #10824-2017073111402747v8).

### Cell isolation from tissues

Lungs were minced and dissociated in RPMI medium containing 10% FCS, 1 mg/ml DNAse I (Sigma-Aldrich), and 7 mg/ml collagenase II (Worthington). Digestion and dissociation were conducted at 37°C in C tubes using the GentleMACS Octo Dissociator (Miltenyi Biotec) according to the manufacturer’s protocol. Cells from spleen and lymph nodes were prepared by mechanical disruption in RPMI medium containing 2% FCS. Red blood cells were lysed using RBC lysis buffer (eBioscience). Single-cell suspensions were filtered through a 100-μm membrane and counted.

### Flow cytometry

Single-cell suspensions were incubated with a mix of fluorescently labeled monoclonal antibodies for 30 min at 4°C. The following antibodies from BD Biosciences, BioLegend, eBioscience, Southern Biotech, and Santa-Cruz Biotechnology were used: anti-CD3 (17A2), anti-CD90.2 (30-H12), anti-CD4 (RM4-5), anti-CD8α (53-6.7), anti-CD6 (J90-462), anti-TCRβ (H57-597), anti-CD44 (IM7), anti-CD25 (PC61), anti-CD5 (53-7.3), anti-CD62L (MEL-14), anti-CD279 (29F.1A12), anti-CD185 (2G8), anti-CD103 (M290), anti-CD39 (24DMS1), anti-CD186 (SA051D1), anti-CD278 (15F9), anti-CD218 (A17071D), anti-CD319 (4G2), anti-CD162 (2PH1), anti-CD200 (OX-90), anti-IA/IE (M5/114.15.2), anti-CD11b (M1/70), anti-CD95 (JO2), anti-CD19 (1D3), anti-CD93 (493), anti-CD38 (90/CD38), anti-IgD (11-26c.2a), anti-IgM (RMM-1), anti-GL7 (GL7), anti-CD138 (281-2), anti-IgG1 (A85-1), anti-IgE (R35-72), anti–IFN-γ (XMG1.2), anti–IL-4 (11B11), anti-Foxp3 (FJK-16s), anti-CD3ε (145-2C11), and anti-CD3ζ (H146-968). Due to the low levels of TCR–CD3 expressed at their surface, CD44^+^ *Lat*^Y136F^ T cells were identified with a combination of CD5 and CD6 or of CD90.2 and CD6 antibodies rather than via CD3 staining. Cell viability was evaluated using DAPI (Life Technologies) or Zombie UV Fixable Viability (BioLegend). For intracytoplasmic staining, cells were fixed and permeabilized using a Cytofix/Cytoperm fixation-permeabilization kit (BD Biosciences). For intranuclear staining, eBioscience Foxp3/Transcription Factor Staining Buffer Set was used according to the manufacturer’s protocol. For cytokines staining, 4 × 10^6^ cells were activated for 4 h at 37°C in 1 ml of 1× Cell Stimulation Cocktail (eBioscience).

Stained cell samples were analyzed on an LSR Fortessa or a FACSymphony Flow Cytometer equipped with FACSDiva software (BD Biosciences), and both instruments were validated prior to data acquisition using Flow Cytometry Calibration Particles (RQC-30-5A; Spherotech). Photomultiplier tube voltages were also adjusted to minimize fluorescence spillover. Single-stain controls were prepared with UltraComp eBeads (Thermo Fisher Scientific) following the manufacturer’s instructions and were used to calculate a compensation matrix. To be able to compare our different experiments, all our data acquisitions were standardized and application settings were recorded. Data were analyzed with either BD FACSDiva V9 Software or FlowJo V10.7 Software (BD Biosciences). For cell sorting, cells were stained as described above and sorted on a FACSAriaIII (BD Biosciences). Lymphocytes were gated based on forward and side scatter, dead cells were excluded using live/dead staining, and doublets were excluded by plotting forward scatter area versus forward scatter height. Among live singlets, T cells were gated as MHCII^−^CD11b^−^CD5^+^CD6^+^ or MHCII^−^CD11b^−^CD6^+^CD90.2^+^ cells.

### Single-cell analysis of flow cytometry data

Data from the acquired FCS files were compensated using FlowJo software (FlowJo LLC). All samples were then analyzed using R package flowCore 1.52.1 (https://bioconductor.statistik.tu-dortmund.de/packages/3.8/bioc/html/flowCore.html). In the following steps, only the channels corresponding to cell surface markers were included and 3,000 cells were randomly selected for each sample. The data were first transformed using parameters automatically calculated with the estimateLogicle function of the flowCore package. We further applied a transformation that scales expression of all markers to values between 0 and 1 using low (1%) and high (99%) percentiles as the boundary. tSNE or UMAP (arXiv:1802.03426) were then used to visualize the high-dimensional data. tSNE was computed using Rtsne package (https://lvdmaaten.github.io/tsne/). tSNE was run on the transformed expression of the cell surface markers with no PCA step and a perplexity equal to 100. UMAP was computed using umapr package available in CRAN. UMAP was run on the transformed expression with the parameter n neighbors set to 30. Finally, the Rphenograph package (Levine et al., 2015) was used for clustering on the transformed expression of the cell surface markers with a number of nearest neighbors set to 150. In the corresponding heatmaps, the values represent the median of transformed cell surface marker expression calculated across cells from all the clusters.

### Flow-cytometry detection of B cells expressing intracellular IgE or IgG1

To specifically detect the IgE or IgG1 that are located in the intracellular organelles of the secretory pathway and avoid caveats linked to the Fc receptors expressed at the B cell surface (Yang et al., 2012), we first blocked the IgE or IgG1 displayed at the B cell surface with an excess of unlabeled monoclonal antibody specific for IgE (R35-72; 553413; BD) or IgG1 (A85-1; 553440; BD). After a washing step, the cells were fixed and permeabilized using a Cytofix/Cytoperm solution (BD Biosciences) and stained in a Perm/Wash buffer (BD Biosciences) containing fluorescently conjugated rat anti-mouse IgE and IgG1 antibodies corresponding to the same clones as those used to block surface IgE or IgG1.

### Tfh-mediated B cell antibody production

In vitro Tfh-mediated B cell antibody production assays were performed as previously described (Sage and Sharpe, 2015). Briefly, Tfh cells, IL4^high^ non-Tfh cells, and latecomer cells were sorted from the spleen of 5-wk-old *Lat*^Y136F^ mice using the key shown in Fig. S5, and CD19^+^ B cells were positively isolated from WT spleens using Dynabeads Mouse CD43 (untouched B cells). Culture involving 3 × 10^5^ CD19^+^ B cells from WT, MHCII^−^, and CD80^−^CD86^−^ mice, and 1 × 10^5^ Tfh cells, IL4^high^ non-Tfh cells, and latecomer cells were set up in 96-well culture plates for 3 d. Cells were then collected for flow-cytometric analysis.

### Cell sorting and RNA preparation for scRNAseq analysis

Cells were isolated from pooled spleens corresponding to seven 2-wk-old and two 5-wk-old WT mice, nine 1-wk-old, 11 1.5-wk-old, six 2-wk-old, five 2.5-wk-old, three 3-wk-old, and one 5-wk-old *Lat*^Y136F^ mice, and T cells were further enriched using the EasySep Mouse T cells Isolation Kit (Stemcell). Each purified T cell sample was then labeled with a specific Hashtag (TotalSeq anti-mouse Hashtag antibody #A0301-A0308; BioLegend), a mix of calibrated TotalSeq-A anti-mouse specific antibodies (CD25, CD39, TCRβ, PD-1, CD103) and a cocktail of six fluorescently labeled anti-mouse antibodies plus a viability dye. CD4^+^ and CD8^+^ cells were then separated as live singlets, lacking CD11b and MHCII molecules and co-expressing CD90.2 and CD6. After sorting, cells were washed in PBS–0.04% BSA as recommended by the 10X Genomics sample preparation protocol and kept on ice until counting was performed. Two to three samples were mixed at a concentration of 1,200 cells/μl and loaded in two to three lanes at a maximum concentration of 12,000 cells per lane and run on a Chromium-controller (10X Genomics). scRNAseq libraries, and hash tag oligonucleotide (HTO) and antibody derived tag (ADT) libraries were generated using the Chromium Single Cell 3′ V3 kit (10X Genomics) according to the manufacturer’s instructions.

### Sequencing and data analysis for scRNAseq analysis

Sequencing was performed by the VIB Nucleomics Core on a NovaSeq6000 Illumina platform. mRNA FASTQ raw files were processed using Cell Ranger v3.0.1 (10X Genomics) software with default parameters to perform alignment, filtering, barcode counting, and unique molecular identifier counting. Reads were aligned to the mouse mm10 genome. Antibodies from ADT and HTO FASTQ raw files were counted using CITE-seq-Count (v1.4.1; https://github.com/Hoohm/CITE-seq-Count). A total number of 53,448 cells were identified with a mean of 48,309 reads per cell and a median of 2,241 genes per cell. Data sets were analyzed using the R package Seurat 3.2.0 (Satija et al., 2015). Cells were first demultiplexed (with the Cell Hashing tags) to their original sample groups using MULTIseqDemux function (with automated threshold finding to define the best quantile). 3,662 doublet cells and 1,103 negative cells were identified and removed. Quality control was performed to remove poor-quality cells. Accordingly, we removed cells with <500 or >6,000 detected genes, cells with more than 15% mitochondrial gene expression, and cells with <15% ribosomal gene expression. Expression data were normalized using the NormalizeData function of the Seurat R package (logNormalize method and scale factor of 10,000). Using PCA (see below), we observed a dependency in number of unique molecular identifiers and number of genes in the first principal component. We centered and regressed the expression data from these factors using the Seurat R package ScaleData function (centering true and scaling false).

PCA was run using Seurat RunPCA function on the 2,000 most variable genes. These genes were identified using the “vst” method of Seurat FindVariableFeatures function. The UMAP was run using Seurat RunUMAP function on the 30 first principal components from PCA. Clustering was done with Seurat FindClusters based on the 30 first principal components from PCA dimensionality reduction and with resolution parameter set to 0.05. Markers for each cluster were identified using Wilcoxon Rank Sum test from Seurat FindAllMarkers with the log fold change threshold set to 0.5. Cell-cycle scores and cell phases were calculated using CellCycleScoring function with s.genes and g2m.genes from the Seurat package. For the analysis of CD4^+^ T cells, a data subsetting was performed on HTO1 to HTO6 (*Lat*^Y136F^ samples) and on clusters 1, 2, 3, 5, and 7 using the Seurat subset function with the following setting: *Cd4* gene expression >0 on the 6 *Lat*^Y136F^ samples restricted to clusters 5, 1, 2, 3, and 7 (Fig. 3). This Seurat object was then computed as above except for the resolution of clustering that was set to 0.75.

### Trajectory inference

Pseudotime analysis was performed on CD4^+^ T cells with Monocle 3 (Cao et al., 2019; Xia et al., 2022) using dimensionality reduction and clusters calculated by Seurat. The principal graph was learned from the data set using default parameters and the CD4^+^ T cells ordered along the graph by selecting cells belonging to the naive CD4^+^ T cell cluster (cluster 1) as a root node. For the sub-branch analysis, cells were manually selected using the choose_graph_segments Monocle function.

### Immunohistochemistry

Spleens and lungs were successively fixed in 4% paraformaldehyde for 3 h at 4°C, washed with PBS, incubated overnight in a PBS-30% sucrose solution, immersed in OCT, and snap-frozen in liquid nitrogen–cooled isopentane. Cryostat spleen sections (10–20 µm thick) were dried in silica beads, washed twice in PBS, and fixed for 45 m at room temperature (RT) using the fixation solution from the eBioscience Foxp3/transcription factor staining buffer set. Sections were subsequently blocked for 1 h at RT in a saturation solution made of the permeabilization solution from the eBioscience Foxp3/transcription factor staining buffer set and containing 2% BSA, 1% goat serum, and 1% FCS. Immediately after blocking, slides were incubated overnight at 4°C with primary antibodies corresponding to anti-mouse B220-FITC (clone RA3-6B2; Thermo Fisher Scientific), anti-mouse CD3ε-PE (clone 145-2C11; BioLegend), and anti-mouse Bcl6-AF647 (clone K112-91; BD Bioscience) in the saturation solution described above. After three washes in PBS, sections were mounted in Fluoromont-G mounting media. Imaging was carried out on an LSM 780 (Zeiss) inverted confocal microscope using a Plan-Apochromat 10 × 0.45 M27.

Cryostat lung sections (10–20 µm thick) were dried in silica beads, washed twice in PBS, and blocked for at least 30 m at RT in a blocking solution (2% BSA, 1% goat serum, and 1% FCS in PBS). Sections were then incubated for 2 h at room temperature with rat anti-mouse CD21/CD35 (clone eBio8D9; Thermo Fisher Scientific) in blocking solution. Slides were washed three times in PBS and then incubated for 2 additional hours at RT with polyclonal goat anti-rat Fab IgG-AF450 (Jackson ImmunoResearch) in blocking solution. After three washes in PBS, sections were incubated overnight at 4°C with anti-mouse CD3-FITC (clone 17A2; BioLegend), anti-mouse B220-PE (clone RA3-6B2; Thermo Fisher Scientific), and anti-mouse EPCAM-AF647 (clone G8.8; Thermo Fisher Scientific) in saturation solution. After a final wash, tissue sections were mounted in Fluoromount-G mounting media. Imaging was carried out on an LSM 780 (Zeiss) inverted confocal microscope using a Plan-Apochromat 20 × 0.8 M27 objective.

### Analysis of confocal images

Images were adapted using the Image J software. Tile-scan spleen and lung pictures were Z-stacked using the maximum intensity projection Z-plan. Contrast and brightness were adjusted for each fluorochrome individually and for each organ the same settings (brightness and contrast values) were applied to sections stained with the same antibody panel to make their fluorochrome intensity comparable.

### Treatment of Lat^Y136F^ mice with CTLA-4-Ig

Recombinant human CTLA-4-Ig (catalog #BE0099) and recombinant human IgG1 Fc control (catalog #BE0096) were purchased from BioXcell. Treatment consisted in injecting 2-wk-old *Lat*^Y136F^ mice intraperitoneally with 10 mg/kg of each recombinant protein, followed by weekly injections.

### Online supplemental material

Fig. S1 shows the flow cytometry analysis of the end-state T cell populations found in *Lat*^Y136F^ spleens. Fig. S2 shows the characterization of the CD4^+^ and CD8^+^ cell clusters described in Fig. 2. Fig. S3 shows the gene signatures corresponding to clusters 3, 5, 7, and 8 identified via scRNAseq analysis of *Lat*^Y136F^ CD4^+^ T cells. Fig. S4 shows the modeling of the differentiation trajectories of *Lat*^Y136F^ CD4^+^ cells. Fig. S5 shows the flow-cytometry gating strategy used to identify the *Lat*^Y136F^ CD4^+^ T cells subsets originally defined via scRNAseq analysis. Data S1 lists the differentially expressed genes corresponding to each of the six CD4^+^ and three CD8^+^ T cell clusters identified during the analysis of *Lat*^Y136F^ spleens at 1, 1.5, 2, 2.5, 3, and 5 wk after birth, and of WT spleens at 2 and 5 wk after birth. Data S2 shows the differentially expressed genes corresponding to each of the 11 CD4^+^ T cell clusters defined during the analysis of *Lat*^Y136F^ spleens at 1, 1.5, 2, 2.5, 3, and 5 wk after birth. Data S3 shows the normalized expression (according to Seurat package) of each of the 1,068 differentially expressed genes in the 11 *Lat*^Y136F^ CD4^+^ T cell clusters defined in Fig. 3.

## Supplementary Material

Data S1 lists the differentially expressed genes corresponding to each of the six CD4^+^ and three CD8^+^ T cell clusters identified during the analysis of *Lat*^Y136F^ spleens at 1, 1.5, 2, 2.5, 3, and 5 wk after birth, and of WT spleens at 2 and 5 wk after birth.

Data S2 shows the differentially expressed genes corresponding to each of the 11 CD4^+^ T cell clusters defined during the analysis of *Lat*^Y136F^ spleens at 1, 1.5, 2, 2.5, 3, and 5 wk after birth.

Data S3 shows the normalized expression (according to Seurat package) of each of the 1,068 differentially expressed genes in the 11 *Lat*^Y136F^ CD4^+^ T cell clusters defined in Fig. 3.

## Data Availability

The data underlying Figs. 2 and 3 are openly available in Gene Expression Omnibus public database under accession number GSE190583.
